# Supplementary figures and images for: Application of optical tweezer technology reveals that PfEBA and PfRH ligands, not PfMSP1, play a central role in Plasmodium falciparum merozoite-erythrocyte attachment
Source: PLoS Pathog. 2024 Sep 23;20(9):e1012041. doi: 10.1371/journal.ppat.1012041 (PMC11449297; doi:10.1371/journal.ppat.1012041)

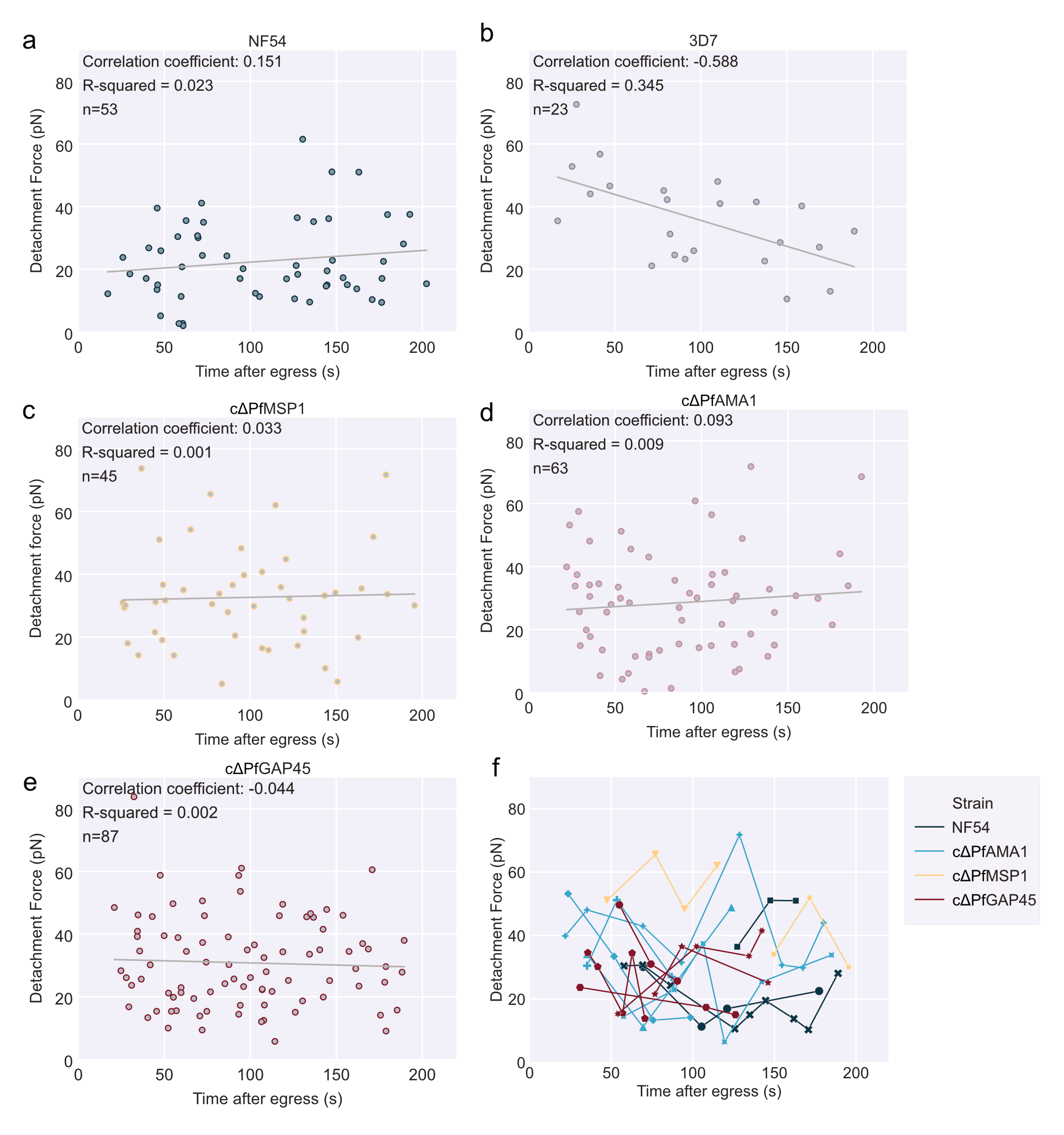

Supplement: S1 Fig — As merozoites are known to lose invasive capacity over time, it is possible that egressed merozoites lose attachment potential/strength over time. All measurements were taken within 180 s of egress, but we nevertheless compared the detachment force and the time post-egress that each measurement was made. No correlation was found between detachment and time post-egress. (a-e) Plot of the time post egress that the detachment force was measured against the measured detachment force, with strain measured above each plot. (c-f) contains data gathered from the inactivated DMSO-treated controls for the three conditional knock-out lines discussed in Fig 2, all made in the 3D7 background. cΔPfAMA1, cΔPfMSP1 and cΔPfGAP45 showed no correlation between time and force (correlation coefficient < 0.1), NF54 showed a very weak positive correlation, correlation coefficient = 0.15 and 3D7 showed a weak negative correlation, correlation coefficient = -0.59, although for this strain there were only 23 points plotted. This indicates that there is no relationship between time post-egress and detachment force. (f) For some merozoites, multiple attachments were made using the same or different erythrocytes. In these cases, the detachment force vs the time post egress for each measurement has been plotted, with lines connecting the attachments of the same merozoite. Only merozoites with more than 2 attachments measured were plotted. Each merozoite is represented by a different style line and marker. This shows that detachment forces do not consistently drop with multiple attachments even after up to 9 measured detachments. (TIFF) [file ppat.1012041.s004.tiff]

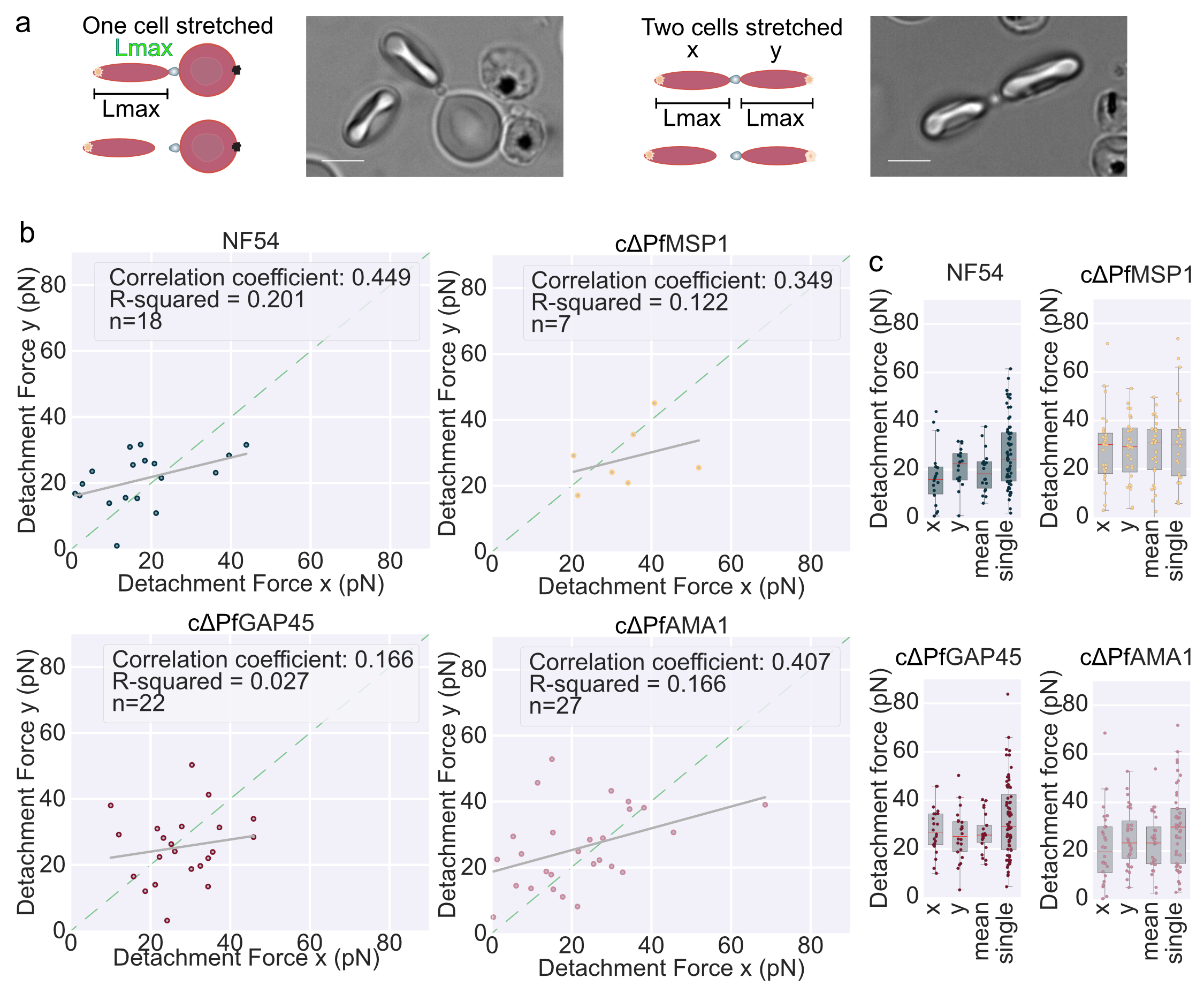

Supplement: S2 Fig — During the optical tweezer manipulation sometimes one erythrocyte becomes attached to the glass slide and only one erythrocyte is stretched by the optical trap, S2 Video, whereas in other cases both erythrocytes are held by optical traps and hence both become stretched, meaning that two forces can be measured for these detachments. We, therefore, compared detachment forces from both erythrocytes and found a positive but weak correlation. This is likely due to biological variation between erythrocytes, as is that we assume that the stiffness constant of all erythrocytes is constant (20 pN/μm); however, there is known to be at least 20% variation between erythrocytes within a single donor [37]. (a) Cartoon shows the two ways that the detachment forces are measured. When a merozoite is attached to two erythrocytes either one erythrocyte is attached to the slide and one trap is used to stretch the other erythrocyte, from which the force is measured, or neither erythrocyte is attached to the slide, meaning traps are used to hold both erythrocytes. In this latter case, both erythrocytes stretch so two measurements are recorded for the detachment. In the rest of the paper in the second case, one force is randomly selected and included, and the other is ignored. Error bar = 5 μm. (b and c) NF54 wild-type data was originally discussed in Fig 1C. For the conditional knockouts cΔPfMSP1, cΔPfAMA1 and cΔPfGAP45 the inactivated DMSO treated controls are plotted, discussed initially in Fig 2. The conditional knockouts are made in the 3D7 background. (b) The force is measured from erythrocytes x and y when two erythrocytes are stretched while attached to the same merozoite. The assignment of a and y to the erythrocytes is random. The grey line shows the fit of the scatter plot. The green line shows the expected fit if x and y were equal. (c) Box plots showing merozoite-erythrocyte detachment force. The central bold line shows the median, with the top and bottom of the box at the 25 [file ppat.1012041.s005.tiff]

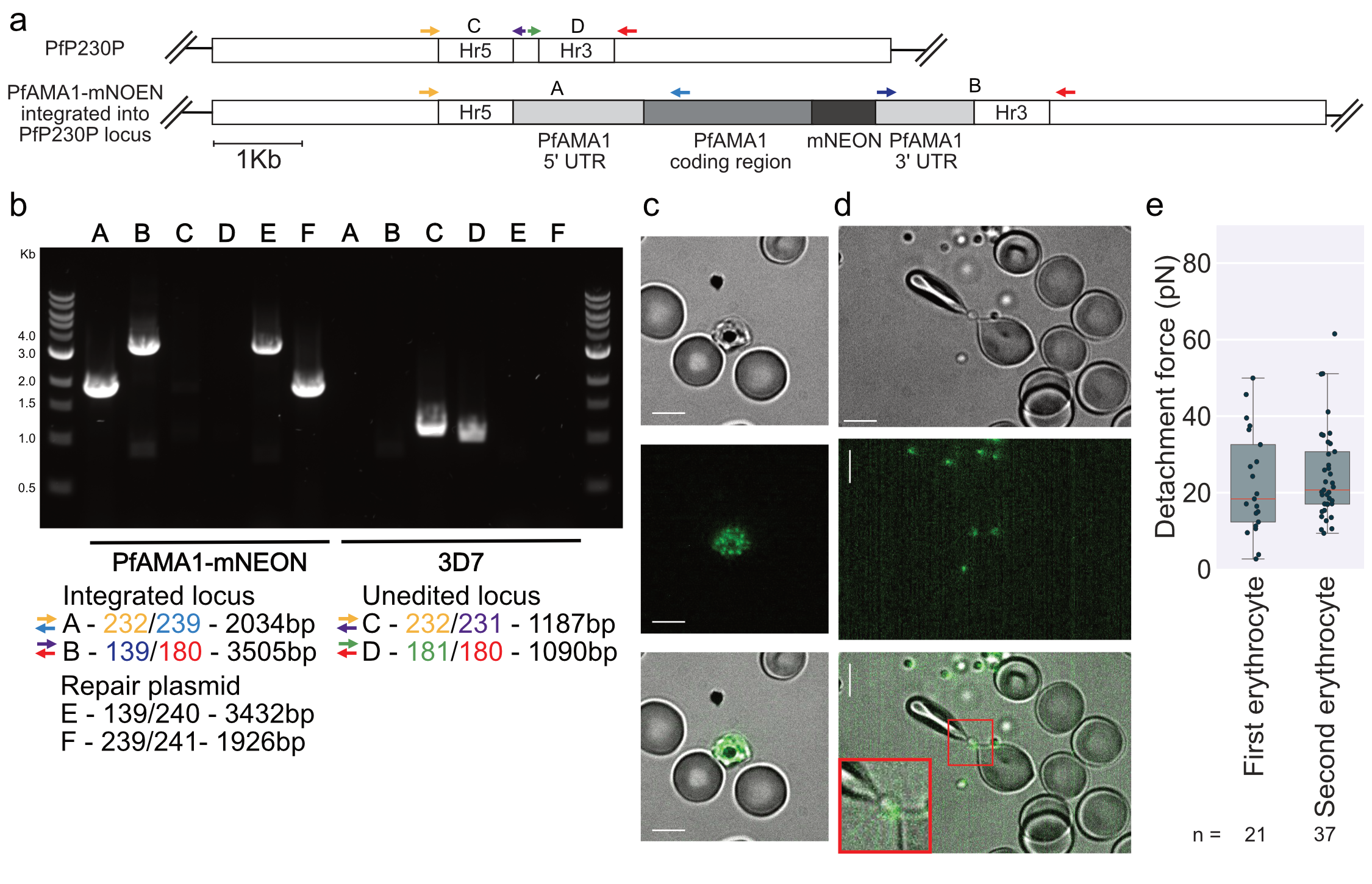

Supplement: S3 Fig — Because merozoites are polar cells it is possible there is a bias to the side that detachment occurs from. To explore this, we attempted to label the apex of the merozoite to see whether detachment predominantly occurs from one end. (a) A line was constructed in the 3D7 background where an additional copy of Apical Membrane Antigen 1 (PfAMA1) C-terminally tagged with the fluorophore mNEON (PfAMA1-mNeon) was integrated into the PfP230P landing site. The diagram represents the genetic structure of the PfAMA1-mNEON line. The top shows the homology regions in the PfP230P gene before PfAMA1-mNEON was integrated. The bottom shows the same region after editing. The sequences for the endogenous PfAMA1 5’UTR and PfAMA1 coding region were linked to the fluorophore mNEONgreen followed by the 3’UTR for the endogenous PfAMA1. The approximate positions of the primers (shown with arrows) to genotype the lines and the letters in between a pair of primers show the reaction ID. (b) Genotyping PCR was used to confirm that the correct edit had been made. The gel for the PCRs run on both the PfAMA1-mNEON line and the unedited parent line 3D7 for reference. The primers used for each reaction are summarised underneath and the sequences are given in S2 Table. Reactions A and B confirm that PfAMA1-mNEON was correctly integrated into the PfP230P locus. C and D confirm the absence of any unedited parasites. Reactions E and F confirm the presence of the homology repair plasmid. PfAMA1-mNeon can also be expressed from the plasmid. (c-d) Show images taken with the same microscope used to carry out all the optical tweezer measurements in this paper. In schizonts PfAMA1 localises to the micronemes and is released at egress, meaning it is initially primarily apically localised, but then diffuses over the merozoite surface over time [100,115]. The left image shows brightfield, the centre image shows the signal from the PfAMA1-mNEON and the right image shows the overlay of the other two. Error bar = [file ppat.1012041.s006.tiff]

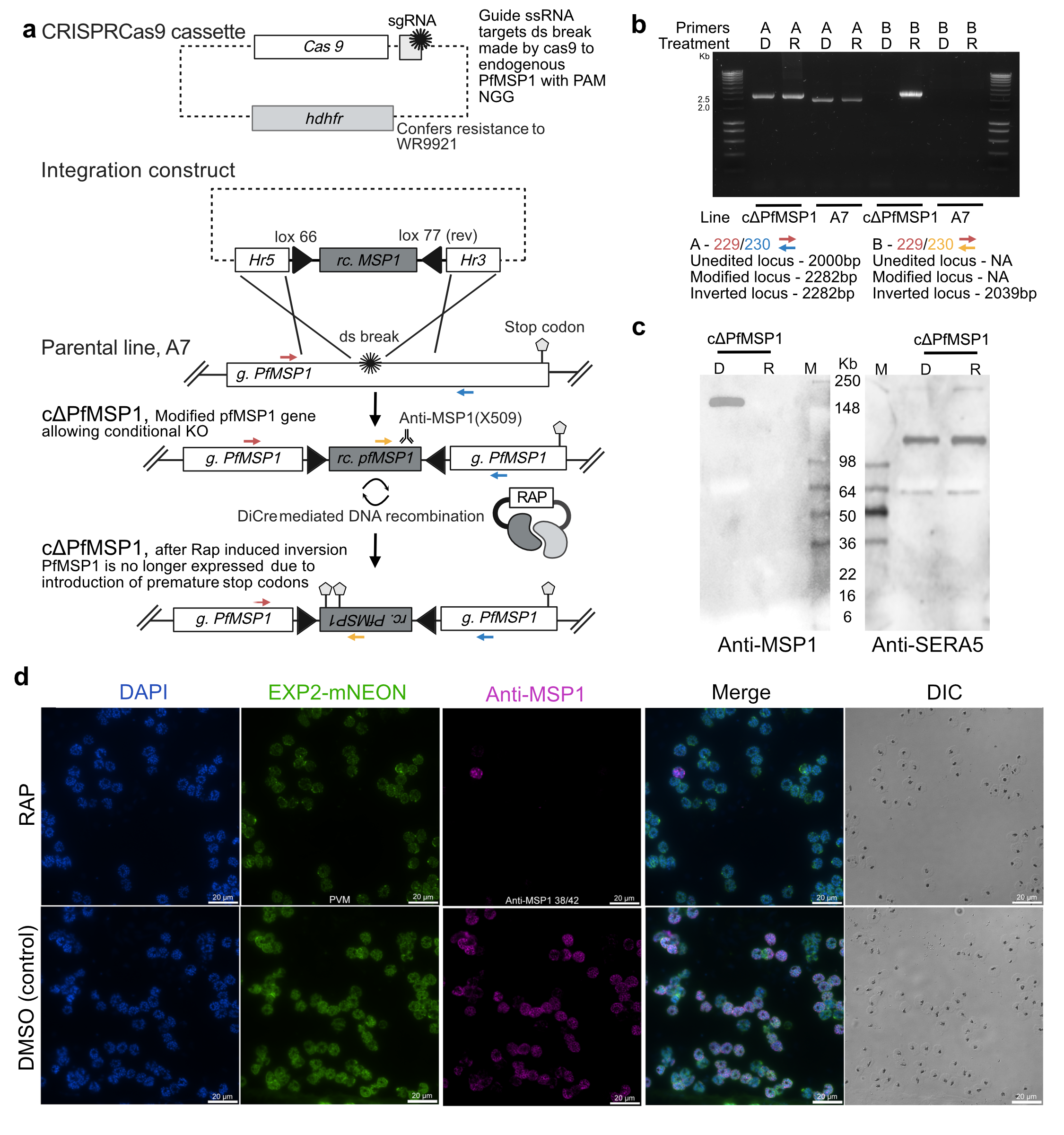

Supplement: S4 Fig — Further characterisation of this line will be published elsewhere. (a) Mutagenesis strategy. The integration construct was designed to integrate re-codonised PfMSP1 (rc. PfMSP1, dark grey) floxed with head-to-head oriented lox66/77 (black, triangles) into the endogenous Pfmsp1 locus (g. PfMSP1) of the parental line A7 which is based on a 3D7 background. Integration is guided by the 5’ and 3’ homology sequence (white, HR5 and HR3). The CRISPR Cas9 cassette co-transfected with the integration construct encodes Cas9 and a guide sgRNA. This ensures a targeted double-stranded break in the endogenous PfMSP1 sequence (PF3D7_0930300), that allows the insertion of the integration construct. The hdhfr gene (light grey) confers resistance to the antifolate WR99210, allowing the selection of transfected parasites. Treatment with rapamycin (rap) activates DiCre, which mediates inversion of the floxed sequence, introducing premature stop codons. Only a ~16 kDa truncated form of PfMSP1 is encoded by the modified locus, and this was not detectable in cells and likely not expressed. The line produced 3D7MSP1KO:lox66/lox71rev is referred to here as cΔPfMSP1. Coloured arrows show the primer binding position. (b) Genotyping gel of PCRs were run to confirm editing. Reaction A (blue arrow, primer-229 and red arrow primer–230) prime off in the endogenous PfMSP1-D sequence demonstrated successful integration at the expected locus (expected product for parental line A7, +/- Rap 2000 bp; expected product after integration, cΔPfMSP1 +/- Rap, 2282 bp). To monitor sequence inversion, an oligo was designed to prime off the integrated inverted recodonised (rc.) sequence (yellow arrow, primer 228)) when paired with the endogenous N-terminal forward primer (red arrow primer 229). Upon the addition of Rap and sequence inversion, a 2039 bp product is expected. No product is expected for DMSO-control treated cΔPfMSP1 parasites and for the parent line (A7) +/- Rap. The sequence of the primers used, are [file ppat.1012041.s007.tiff]

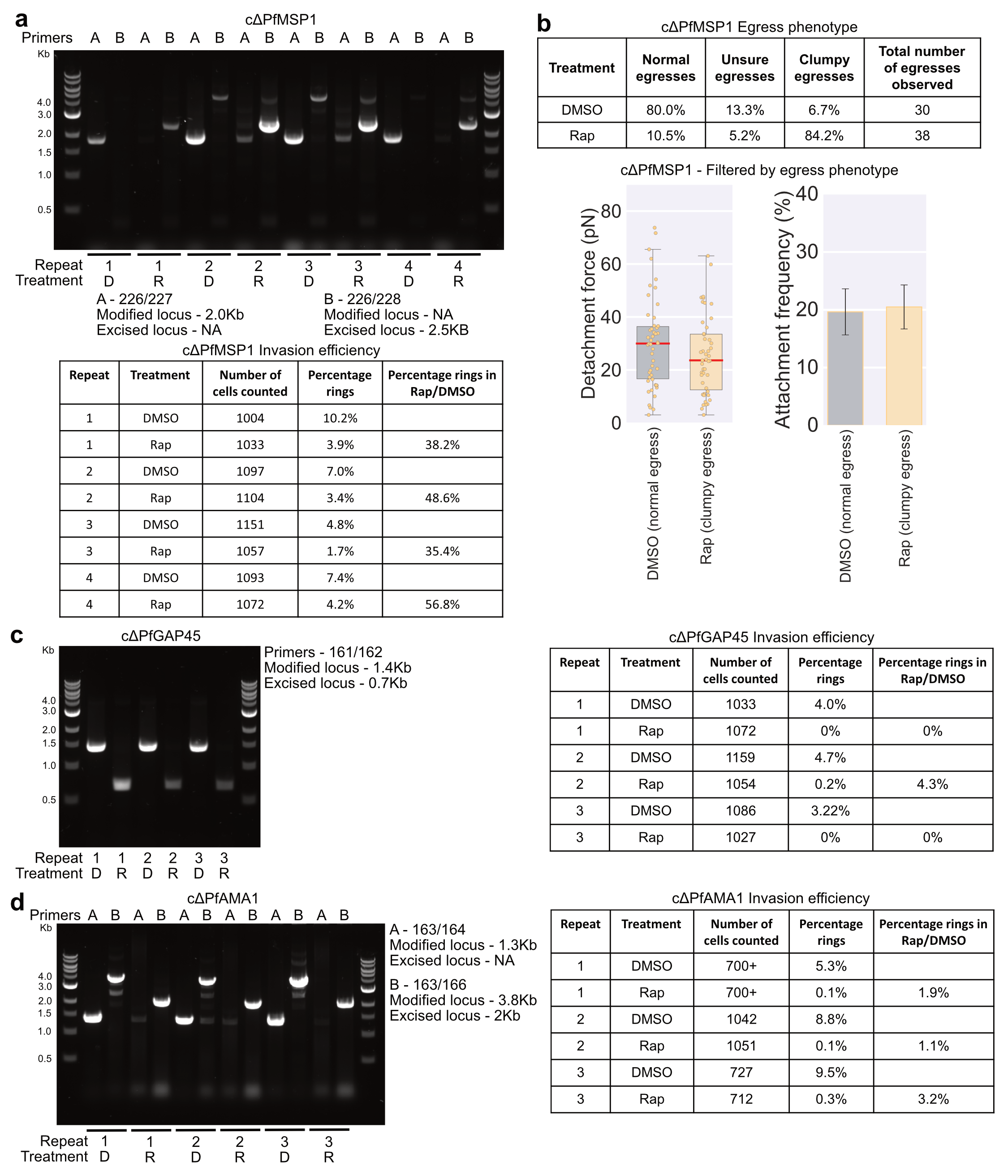

Supplement: S5 Fig — Validation of conditional knock-out lines used of optical tweezer experiments (a, c-d) Rapamycin-induced deletion was validated for all three-conditional knock-out lines tested in Fig 2 at the same time as imaging was carried out. Samples of rapamycin-treated (Rap) and DMSO-control (the solvent in which rapamycin is dissolved) treated parasites were collected and washed in PBS, and genotyping PCR was used to confirm gene excision. All gels are for genotyping PCRs, indicated are the primers used, sequences are given in S2 Table as well as the expected sizes for the bands in the modified locus (locus with integrated loxP sites) and the excised locus that should be present after rapamycin treatment. The numbers indicate the repeat number. The ladder is NEB 1 kb DNA Ladder (N0552). Tables show a summary of the parasitimia assessed at the beginning of the next cycle after DMSO/Rap addition by counting the number of rings in Giemsa smears. (a) Measurements were collected for the cΔPfMSP1 line. (b) For cΔPfMSP1 egresses were assessed during the optical tweezer assays and classified as normal, clumpy or unsure (for egresses that we were not sure if they were clumpy as they were hard to distinguish). The table summarises what fraction of the egresses fell into each class. Box plot showing the detachment force of merozoite-erythrocyte attachment. The central bold line shows the median, with the top and bottom of the box at the 25th and 75th percentiles and the whiskers showing the total range of the data. The bar chart shows the frequency of positioned cells that lead to attachment. The distributions were compared with a t test, and there were no significant differences at a 5% level of significance. Error bars show the SEM. (c) Measurements were collected for the cΔPfAMA1 line. (d) Measurements were collected for the cΔPfGAP45 line. (TIFF) [file ppat.1012041.s008.tiff]

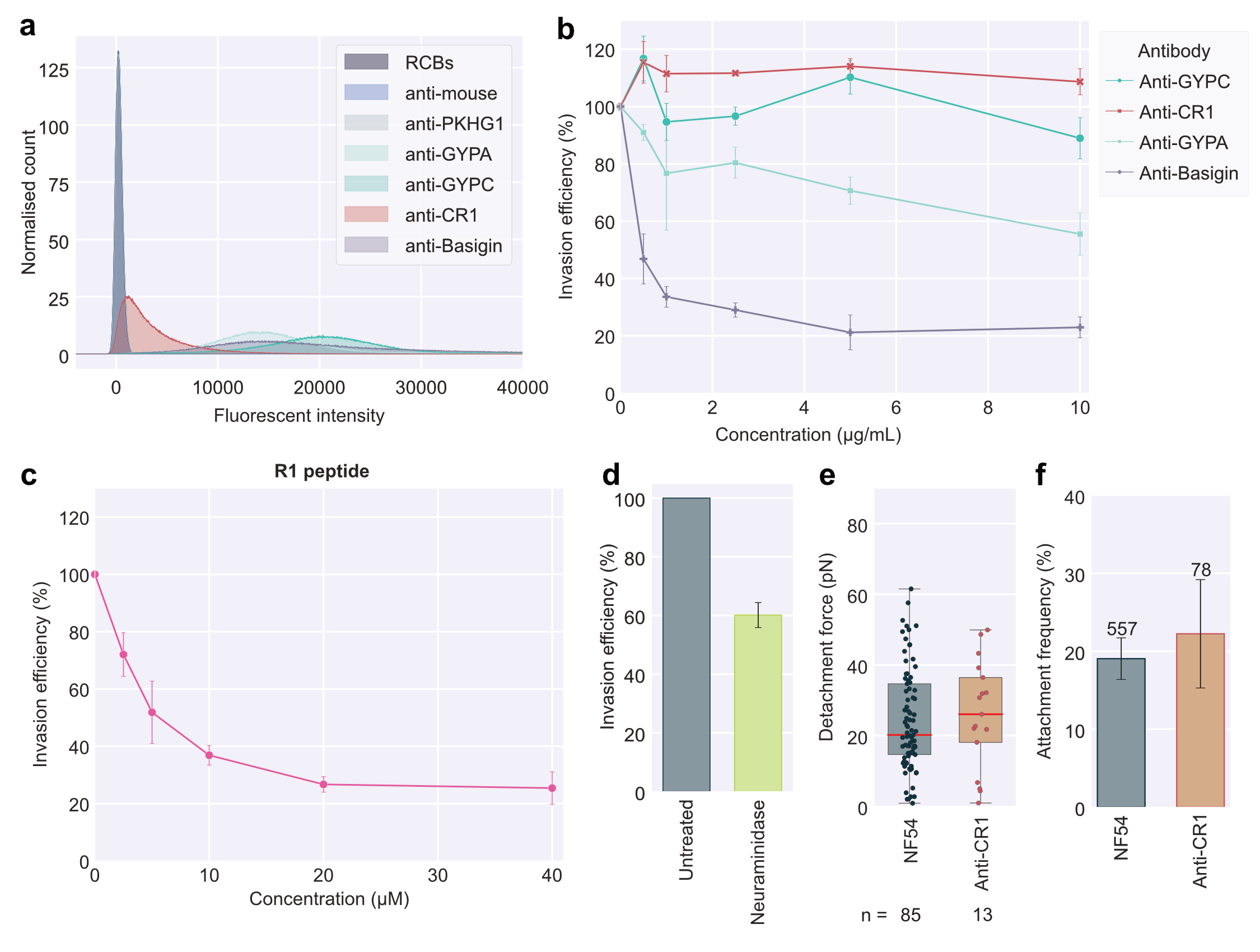

Supplement: S6 Fig — (a) Validation of antibody binding to erythrocytes. Primary antibodies used were anti-CR1 (Ab25 –E11), anti-Basigin (AB119114 MEM-M6/6), anti-GYPA (BRIC 256), anti-GYPC (BRIC 4) and control antibody (which should not bind erythrocytes) anti-PKHG1 (ab121979). Erythrocytes were incubated with each of the primary antibodies, and then a secondary anti-mouse Alexa Fluro plus 488 conjugated antibody was used to detect binding in flow cytometry. Data was collected for three independently labelled wells for blood from two donors. The area under each histogram was normalised to have the same area and then averaged for each condition. The erythrocytes (unlabelled), anti-mouse secondary antibody and the control anti-PKHG1 all showed negligible binding, whereas the histograms for the anti-CR1, anti-Basigin, anti-GYPA and anti-GYPC all showed increased fluorescence intensity relative to controls, indicating all primary antibodies used were able to bind to the erythrocytes. (b-d) Growth inhibition assay done using wild-type strain NF54. When no treatment was present, a 100% invasion efficiency was set as the percentage of invaded erythrocytes. Error bars show the standard error of the mean (SEM). (b) Investigates the effect of antibodies, anti-GYPC data based on two repeats. (c) Investigates the effect of the R1 peptide inhibitor. (d) Investigating the effect of invasion into blood treated with 66.7 mU/ml neuraminidase, mean invasion efficiency was 60 ± 4%, based on two repeats. (e) Anti-CR1 E11 binds erythrocytes (a) but has no detectable effect on invasion, even at the highest concentration of 10 μg/ml (b). However, we still tested for an effect on attachment with the optical tweezers. Only one biological repeat was done for NF54+anti-CR1 antibody. Shown is a box plot showing the detachment force of merozoite-erythrocyte attachment. Mean detachment force NF54 24 ± 2 pN; NF54+anti-CR1 26 ± 4 pN, not significant compared to NF54 (t-test p = 0.66). The central bold line shows the [file ppat.1012041.s009.tiff]

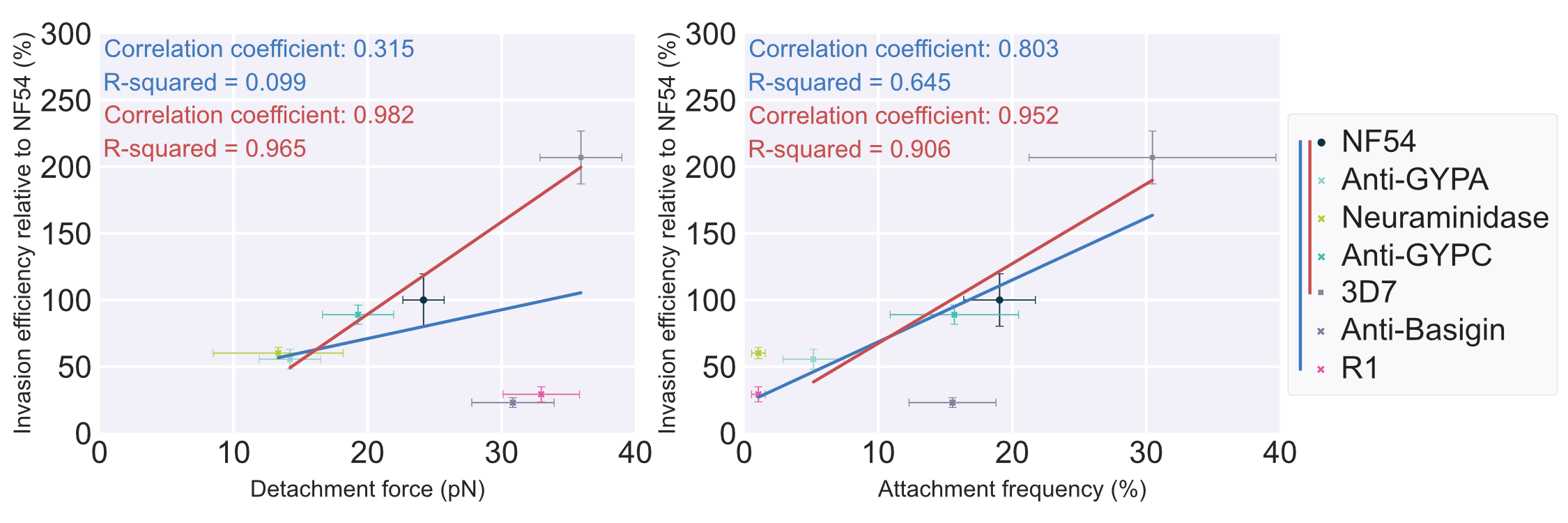

Supplement: S7 Fig — Correlation of the invasion efficiency relative to wild-type line NF54 (based on measurements in Figs 1E, S5B and S5C) to the detachment force (Figs 1C and 2C) and attachment frequency (Figs 1D and 2C) for the wild-type line NF54, 3D7 and NF54 in the presence of 10 μg/ml of antibodies anti-GYPC (BRIC 4), anti-GYPA (BRIC 256) or anti-Basigin (MEM-M6/6); or with neuraminidase treatment 66.7 mU/ml erythrocytes or 20 μM R1 peptide that inhibits PfAMA1-PfRON2 binding. The grey line and text show the correlation of all the data points. The red line shows the correlation of NF54, Anti-GYPA, neuraminidase, anti-GYPC and 3D7, excluding the inhibitors that target late-in invasion (R1 and anti-basigin) and so likely affect invasion after attachment. Error bars show the standard error of the mean (SEM). (TIFF) [file ppat.1012041.s010.tiff]

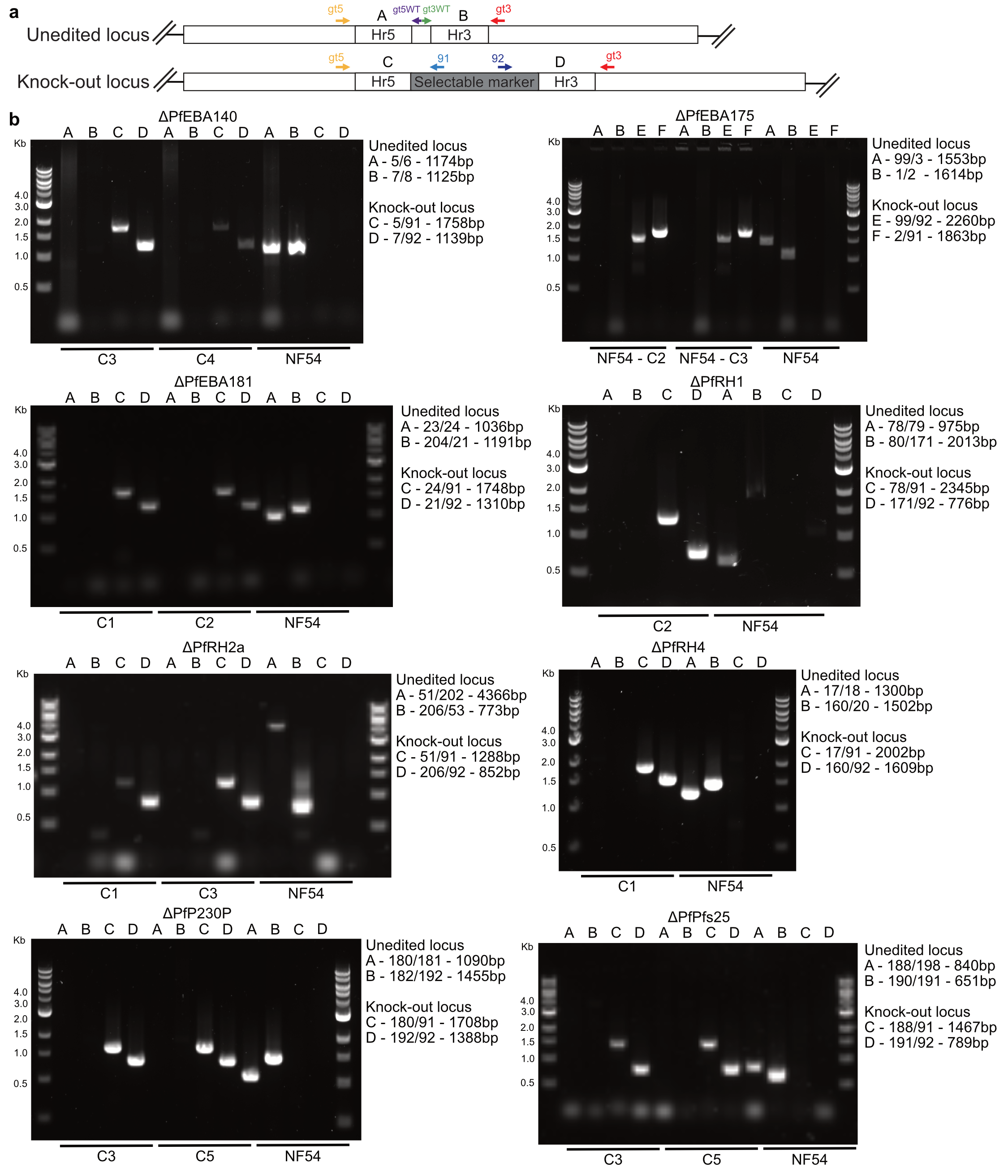

Supplement: S8 Fig — (a) Schematic to represent the genetic change made in the knock-out lines. The red arrows show the approximate positions of the primers used to genotype the lines and the letters in between a pair of primers refer to the reactions shown in the panels below. (b) Show gels for genotyping PCRs; details of the primers used are shown to the side as well as the expected sizes for the bands in the unedited locus and the locus after editing; primer sequences are given in S2 Table. The only knock-out with a different structure is ΔPfEBA175, as the selectable marker was introduced in the opposite direction, so the primers that bind to the selectable marker were used in the opposite pairs: E–forward primer in front of HR5 and 92 and F– 91 and the reverse primer next to HR3. For each gene, the primers were tested with samples of the knock-out clones used for phenotyping along with a sample of NF54 as a control for what the bands for the unedited locus look like. The ladder is NEB 1 kb DNA Ladder (N0552). (TIFF) [file ppat.1012041.s011.tiff]

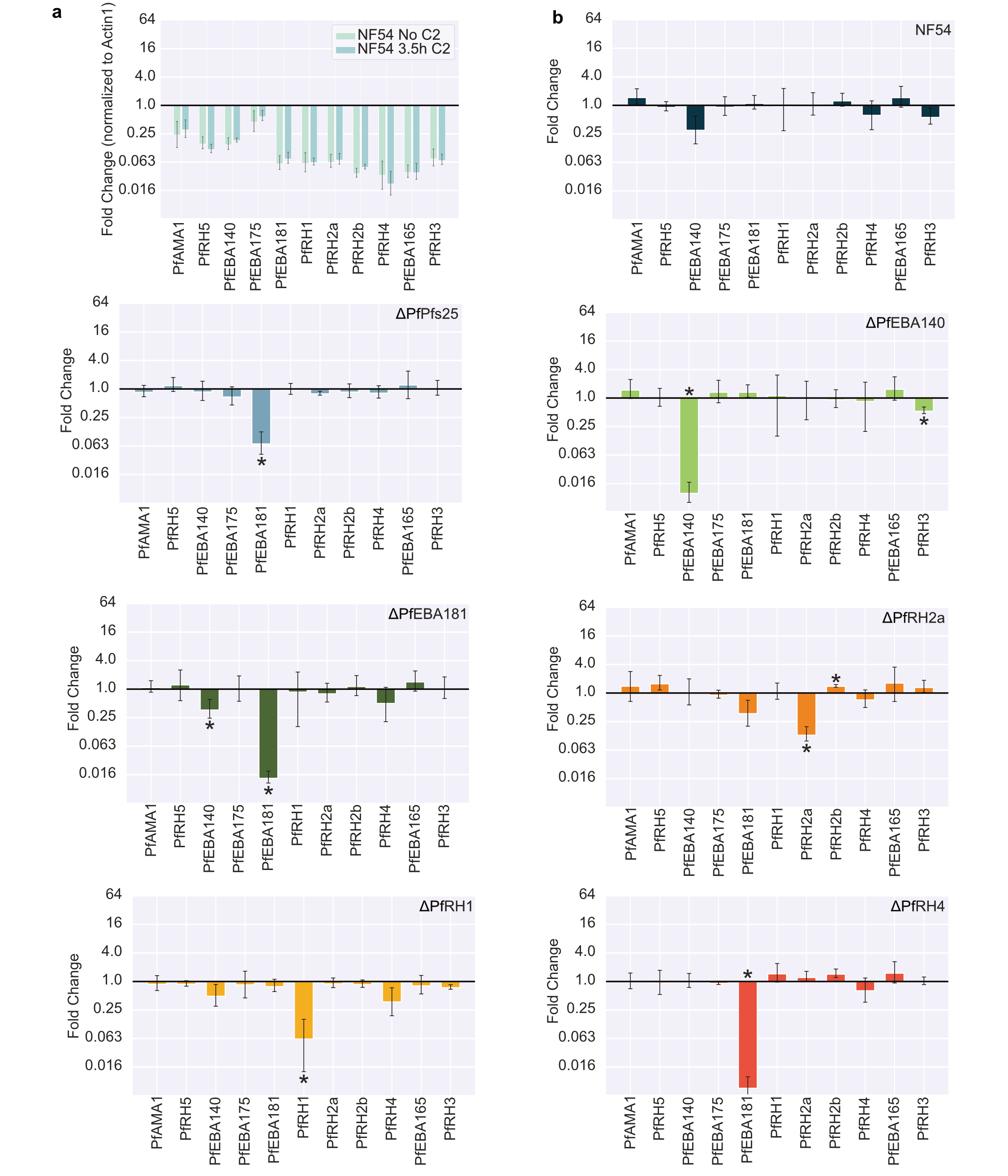

Supplement: S9 Fig — Error bars show the standard deviation between repeats. (a) Comparison of gene expression in samples collected from tightly synchronised NF54 line both before C2 treatment (green) and 3.5 h after C2 treatment (blue). Data represents the mean from three samples collected on different days (biological replicates), and expression is plotted as the fold change relative to the PfActin I housekeeping gene in the sample. Gene expression was very similar before and after compound 2 treatment. A 3.5 h C2 treatment was used for the collection of all other samples. (b) Shows the expression profiles for invasion genes in the knock-out lines (the gene deleted in each line is indicated above the graphs), Cq values were interpolated to a standard curve of genomic DNA, with expression in each sample normalised to the housekeeping gene PfActin I and presented as fold-change relative to the expression of those same genes in the control NF54ΔPfP230P line. Four samples for each line were collected (biological replicates collected with invasion into different blood), the samples for all lines were collected in parallel. For each sample triplicate wells were run (technical replicates). NF54 showed no significant differences in any gene tested relative to ΔPfP230P. Across all the lines tested, there was no significant change in PfAMA1 and PfRH5 expression, as expected as they are both essential genes and are not known to be variably expressed; this also confirms that the samples were all consistently synchronised. The control line NF54ΔPfs25 showed significant 14-fold downregulation of PfEBA181 relative to NF54ΔPfP230P (t-test p = 0.0012; interestingly, this was the line that showed significantly ~30% higher invasion rates compared to NF54 and NF54ΔPfP230P, Fig 3A). The NF54ΔPfRH4 line showed an even larger 179-fold downregulation of PfEBA181 expression (t-test p = ≤0.0000) (the Cq values were close to the no reverse transcriptase controls for all samples tested, meaning an almost absence [file ppat.1012041.s012.tiff]

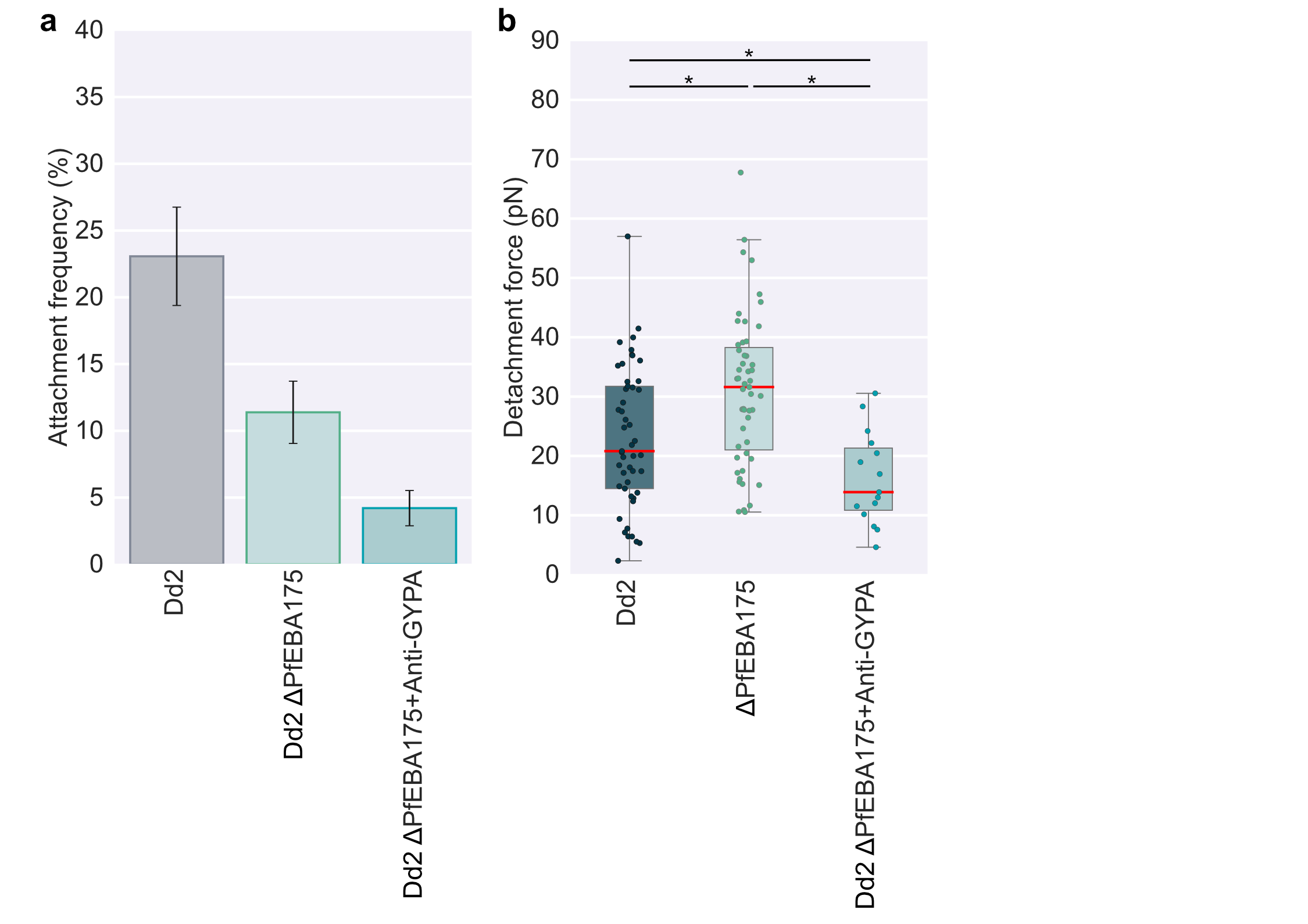

Supplement: S10 Fig — The antibody anti-GYPA (BRIC 256) was used at 10 μg/ml. (a) The bar chart shows the frequency of erythrocyte-merozoite-erythrocyte positions that lead to attachment of the merozoite to both erythrocytes. (b) The box plots show the measured detachment forces. The central bold line shows the median, with the top and bottom of the box at the 25th and 75th percentiles and the whiskers showing the total range of the data. Error bars show the SEM. (TIFF) [file ppat.1012041.s013.tiff]
