# Supplementary material for: Application of optical tweezer technology reveals that PfEBA and PfRH ligands, not PfMSP1, play a central role in Plasmodium falciparum merozoite-erythrocyte attachment
Source: PLoS Pathog. 2024 Sep 23;20(9):e1012041. doi: 10.1371/journal.ppat.1012041 (PMC11449297; doi:10.1371/journal.ppat.1012041)
Supplement: S1 Table — (DOCX) [file ppat.1012041.s014.docx]

| **Binds** | **Version/epitope** | **Type** | **Supplier or reference** | **Product code** |
| --- | --- | --- | --- | --- |
| CD55 | Targets amino acids 71-300 | Rabbit polyclonal | Abcam | Ab96680 |
| CD147/Basigin | MEM-M6/6 | Mouse monoclonal | Abcam | Ab119114 |
| GYPA | Similar to LICR/LON R18 that binds amino acids 49-52 | Mouse monoclonal | IBGRL | BRIC 256 |
| CR1 | E11 | Mouse monoclonal | Abcam | ab25 |
| GYPC | Binds extracellularly near the N terminus | Mouse monoclonal | IBGRL | BRIC 10 |
| GYPC | Binds around amino acids 2-21 | Mouse monoclonal | IBGRL | BRIC 4 |
| PKHG1 |  | Rabbit polyclonal | Abcam | Ab121979 |
| Anti-mouse | Mouse IgG | Goat – conjugated to Alexa Fluro plus 488 | Invitrogen | A32723 |
| MSP1 | X509 p38/42 | Human Monoclonal | (1) |  |
| SERA5 |  | Rabbit Polyclonal | (2) |  |
| Anti-Human |  | Goat HRP conjugated | Abcam | ab6858 |
| Anti-Rabbit |  | Goat HRP conjugated | Abcam | ab205718 |
| Anti-Human |  | Goat alexofluor647 conjugated | Life technologies, Invitrogen | A-21445 |

**References**

1. Blackman MJ, Whittle H, Holder AA. Processing of the Plasmodium falciparum major merozoite surface protein-1: identification of a 33-kilodalton secondary processing product which is shed prior to erythrocyte invasion. Mol Biochem Parasitol [Internet]. 1991 Nov;49(1):35–44. Available from: https://linkinghub.elsevier.com/retrieve/pii/016668519190128S

2. Stallmach R, Kavishwar M, Withers‐Martinez C, Hackett F, Collins CR, Howell SA, et al. Plasmodium falciparum SERA5 plays a non-enzymatic role in the malarial asexual blood-stage lifecycle. Mol Microbiol [Internet]. 2015 Apr 11;96(2):368–87. Available from: https://onlinelibrary.wiley.com/doi/10.1111/mmi.12941
