# Supplementary material for: Application of optical tweezer technology reveals that PfEBA and PfRH ligands, not PfMSP1, play a central role in Plasmodium falciparum merozoite-erythrocyte attachment
Source: PLoS Pathog. 2024 Sep 23;20(9):e1012041. doi: 10.1371/journal.ppat.1012041 (PMC11449297; doi:10.1371/journal.ppat.1012041)
Supplement: S2 Table — (DOCX) [file ppat.1012041.s015.docx]

| **Primer number** | **Name** | **Sequence** | **Use** |
| --- | --- | --- | --- |
| 1 | P0001_0731500gt3WT | ggctcatggggaaataagtgaac | Genotyping of Knock-out lines |
| 2 | P0002_0731500_gt3 | gcattcaaccttcagagtctctatc | Genotyping of Knock-out lines |
| 3 | P0003_0731500gt5WT | tgcataattggattctacgatcagg | Genotyping of Knock-out lines |
| 5 | P0005_1301600_gt5 | tccgaaaatgaagataattctgg | Genotyping of Knock-out lines |
| 6 | P0006_1301600gt5wt | aatcttcctcatgaccacgatg | Genotyping of Knock-out lines |
| 7 | P0007_1301600_gt3 | cctgaattatcactagaatgtgaac | Genotyping of Knock-out lines |
| 8 | P0008_1301600gt3WT | aaaggatgaaaatgcattgtgtag | Genotyping of Knock-out lines |
| 17 | P0017_0424200_gt5 | ttgatgaattaatacctatcacacaac | Genotyping of Knock-out lines |
| 18 | P0018_0424200gt5WT | ctaagggagtggttattataattttgg | Genotyping of Knock-out lines |
| 20 | P0020_0424200gt3WT | ccaaaattataataaccactcccttag | Genotyping of Knock-out lines |
| 21 | P0021_0102500_gt3 | ccatgttcctgtacttttaaattttcagg | Genotyping of Knock-out lines |
| 23 | P0023_0102500gt5WT | cctaatatagagacctcatatctttgtc | Genotyping of Knock-out lines |
| 24 | P0024_0102500_gt5 | tgaaagggaaaatgaatatgtgtttg | Genotyping of Knock-out lines |
| 51 | P0051_1335400Fgt5 | gaaatattgaaaacgtctattaaccc | Genotyping of Knock-out lines |
| 53 | P0053_1335400gt3WT | acaagagcaagaaagactgg | Genotyping of Knock-out lines |
| 78 | P0078_0402300_gt5 | ttttgcttattcttatttgttaaaatatgc | Genotyping of Knock-out lines |
| 79 | P0079_0402300gt5WT | tttgtgataatctttatccttcgg | Genotyping of Knock-out lines |
| 80 | P0080_0402300gt3WT | ggaaatgattagaatccattcgg | Genotyping of Knock-out lines |
| 82 | P0082_EBL1_amp_F | agattttataaaaatttttattagaacgacg | Amplify region for sequencing to confirm EBL1 5 thymidine insertion |
| 83 | P0083_EBL1_seq_1_F | ctattaatgtcaagataaatatatctatacgg | Sequence amplified EBL1 region to confirm 5 thymidine insertion |
| r84 | P0084_EBL1_seq_2_R | tgtattcgtcttattggggc | Sequence amplified EBL1 region to confirm 5 thymidine insertion |
| 85 | P0085_EBL1_midamp_R | tccactcctctatccatcg | Amplify region for sequencing to confirm EBL1 5 thymidine insertion |
| 91 | P0091_P7 | tttatcatgcacattggaataatac | Genotyping of Knock-out lines in insert |
| 92 | P0092_P8 | atttatcttttacaatatgaacataaagtacaac | Genotyping of Knock-out lines in insert |
| 93 | P0093_hr5_colPCR_F | tctcgagtttattcgaaatgtgg | pCC1 colony PCR |
| 94 | P0094_hr3_colPCR_R | aaataccgcacagatgcg | pCC1 colony PCR |
| 99 | P0099_0731500_gt5 | acgtcattccataaaaacaagg | Genotyping of Knock-out lines |
| 139 | P0139_qPCR_R_AMA1 | cataatctgttaaatgttgttcatattgtttaggttgat | AMA1-mNEON genotyping |
| 160 | P0160_0424200gt3v2 | cgttatctttccctgaagattttcc | Genotyping of Knock-out lines |
| 161 | P0161_GAP45_int_F2 | tgtttaatacatactgtgtaatcctt | Genotyping of PfGAP45 condition knock-out line |
| 162 | P0162_GAP45_R1 | ccaagatcacatccacatcttcttga | Genotyping of PfGAP45 condition knock-out line |
| 163 | P0163_AMA1_int_F1 | gtgatgtgtatcgtccaatc | Genotyping of PfAMA1 condition knock-out line |
| 164 | P0164_AMA1_Int_R | ggatgagacagggcagtagtcgc | Genotyping of PfAMA1 condition knock-out line |
| 166 | P0166_end_dsB361_R | gtgatgctcttttttcttcgcccc | Genotyping of PfAMA1 condition knock-out line |
| 171 | P0171_0402300_gt3bR | atatttttccttgatgtgataaaatcc | Genotyping of Knock-out lines |
| 180 | P0180_0208900_gt5 | cttatgaacgtacatcaggagaag | Genotyping of Knock-out lines and AMA1-mNEON genotyping |
| 181 | P0181_0208900gt5WT | tcatatggacacataatacctacaaattc | Genotyping of Knock-out lines and AMA1-mNEON genotyping |
| 182 | P0182_0208900gt3WT | agaagagtgtactataagaagcgatggag | Genotyping of Knock-out lines |
| 188 | P0188_1031000_gt5 | ttatagagtttggctagatatataagg | Genotyping of Knock-out lines |
| 189 | P0189_1031000gt5WT | cctatattacatgagcaaactcc | Genotyping of Knock-out lines |
| 190 | P0190_1031000gt3WT | aaaccaaatgctcattaaaatgc | Genotyping of Knock-out lines |
| 191 | P0191_1031000_gt3 | catgacaaaattttacttagttttatttacc | Genotyping of Knock-out lines |
| 192 | P0192_208900gt3v2 | tttaccgaagtatcaaaagatatgg | Genotyping of Knock-out lines |
| 202 | P0202_1335400gt5W2 | gttgttctctttctgctaactcg | Genotyping of Knock-out lines |
| 204 | P0204_0102500gt3W2 | tgaaaagaaagaatttaaaccatgg | Genotyping of Knock-out lines |
| 206 | P0206_1335400_2gt3 | taaagagaacatcatcattcgg | Genotyping of Knock-out lines |
| 226 | P0226_3D7 Endo R2 | ccaggactggatgtagatgatg | Genotyping of PfMSP1 condition knock-out line |
| 227 | P0227_MSP1 recod F2 | gaggaggcccataatcttattagtg | Genotyping of PfMSP1 condition knock-out line |
| 228 | P0228_MSP1 recod R2 | cactaataagattatgggcctcctc | Genotyping of PfMSP1 condition knock-out line |
| 229 | P0229_HA1_F2 | ggtacaagtccatcatctcgttcaaacac | Genotyping of PfMSP1 condition knock-out line |
| 230 | P0230_3D7 Endo R1 | ggtggtgatggttgtgttggtgg | Genotyping of PfMSP1 condition knock-out line |
| 231 | P0231_p230p genotype WT F | agaagagtgtactataagaagcgatggag | AMA1-mNEON genotyping |
| 232 | P0232_p230p genotype Flank R | catgtgatttagtattaataactttaacttgatc | AMA1-mNEON genotyping |
| 233 | P0233_AMA1 3'UTR R | agtatctgaaacactatccttatcaacgtaatttgtctgcattcttt | AMA1-mNEON genotyping |
| 239 | P0239_AMA1_3'_UTR_F | aatgtgaactataataatttcaacgtct | AMA1-mNEON genotyping |
| 240 | P0240_seq_pcc1_NotI | cacacaggaaacagctatgaccatg | AMA1-mNEON genotyping |
| 241 | P0241_ScreenRev | atgcttaagacagatcttcggac | AMA1-mNEON genotyping |
| 242 | P0242_AMA1 F | ttgggaatccaatagaaggttgcgtgcatgccatttaagtattaatg | AMA1-mNEON genotyping |
| 243 | P0243_AMA1 R | atagtatggtttttccatcagaactg | AMA1-mNEON genotyping |
| 244 | P0244_mNeonGreen AMA1 F | agttctgatggaaaaaccatactatatggttagcaagggcgaagaag | AMA1-mNEON genotyping |
| 245 | P0245_mNeonGreen AMA1 R | acgttgaaattattatagttcacattttatttataaagctcgtccat | AMA1-mNEON genotyping |
