# Supplementary material for: Application of optical tweezer technology reveals that PfEBA and PfRH ligands, not PfMSP1, play a central role in Plasmodium falciparum merozoite-erythrocyte attachment
Source: PLoS Pathog. 2024 Sep 23;20(9):e1012041. doi: 10.1371/journal.ppat.1012041 (PMC11449297; doi:10.1371/journal.ppat.1012041)
Supplement: S3 Table — Table summarising the sequences used in constructing the liens made for this paper. This includes the two guide sequences used (guide 1 and guide 2). The start and end sequences of the homology regions that were used are listed which are the sequences used as the overhand in the Gibson assembly (Hr5_F, Hr5_R, Hr3_F and Hr3_R). Finally, the sequences are given for the genotyping primers used to confirm correct integration in the knock-out lines (Gt5, Gt5WT, Gt3 and GT3WT). (DOCX) [file ppat.1012041.s016.docx]

| **Line** | **Gene ID** | **Guide sequence** | | **Homology regions** | | | |
| --- | --- | --- | --- | --- | --- | --- | --- |
|  |  | **Guide 1** | **Guide 2** | **Hr5_F** | **Hr5_R** | **Hr3_F** | **Hr3_R** |
| ΔPfP230P | PF3D7_0208900 | gaatattattctaatgataa | caagaatttcttaatcatgt | catatttatgtgacttcttaaacaaaaa | caaccttctattggattccca | gttgataaggatagtgtttcagat | ggattaatattccca |
| ΔPfs25 | PF3D7_1031000 | gaatgtaagaatgtaacttg |  | gtcgtttttataaacttcaatatagatttt | ccaagattacatttacaagcgt | cctgtaaagctgttgatgga | ccaagttgatatctttctgaatttac |
| ΔPfEBA140 | PF3D7_1301600 | cataaagtttgtcttctggg | agaagacaaactttatgtct | aaactcacgtttagaaacaa | tgcaatatctacatcatttt | cgtgaaaaatggtgggatga | tgtgcattcgttgttctat |
| ΔPfEBA181 | PF3D7_0102500 | tattaatagacaaagatatg | aaaagaaagaatttaaacca | aagagtatttgaaggaaagg | cactcattattacatccggt | ggtgtatgtgtgtcaccgag | ctttgtcagttcgtacggtt |
| ΔPfRH1 | PF3D7_0402300 | ttatttagaagcaacaaaag | atattaaatgagtttcatca | gattttctgcaacattgttt | gcataatcatactgcatagt | cgattcaacttatttctgga | gcgaatttccttttccttag |
| ΔPfRH2a | PF3D7_1335400 | aagacaagagcaagaaaggc | aagtaaactagaatctgata | gttctaaagaaagaggaaat | gttgtttcttaatctcatct | ggatgaactaacaaaagaaa | ccactattagtctttataag |
| ΔPfRH2b | PF3D7_1335300 | agtcatagaagtaacaccca | catgactactagcatcacgt | gttctaaagaaagaggaaat | gttgtttcttaatctcatct | ttggtagcgacattcatatg | ccagcattatatacgaaatc |
| ΔPfRH4 | Pf3D7_0424200 | aatgtacaaaactaagggag | tattgaatgtacaaaactaa | caaaacaaatctggaaaaca | cgtctacatcctttttgatt | ggaaaacacaaaaatcaaca | tctggaagttccccttttac |
| AMA1-mNEON | (P230P) - PF3D7_0208900 | gaatattattctaatgataa |  | gtgacactatagaatactcgcggccgccatatttatgtgacttcttaaac | caaccttctattggattcccaaaagatt | gttgataaggatagtgtttcagatactaatg | cggactagtcctccccgcggggattaatattcccattaggacaat |
