# Supplementary material for: Application of optical tweezer technology reveals that PfEBA and PfRH ligands, not PfMSP1, play a central role in Plasmodium falciparum merozoite-erythrocyte attachment
Source: PLoS Pathog. 2024 Sep 23;20(9):e1012041. doi: 10.1371/journal.ppat.1012041 (PMC11449297; doi:10.1371/journal.ppat.1012041)
Supplement: S4 Table — F—forward, R—reverse. Ref—reference sequence originally published in. Length—Length of PCR. (DOCX) [file ppat.1012041.s017.docx]

| **Proteins** | **Gene ID** | **F primer** | **R Primer** | **Forward** | **Reverse** | **Source** | **Length** | **Relationship to KO** |
| --- | --- | --- | --- | --- | --- | --- | --- | --- |
| Actin1 | PF3D7_1246200 | P0150_qPCR_F_Actin | P0151_qPCR_R_Actin | TGCACCACCAGAGAGAAAAT | ACTTGGTCCTGATTCATCGT | (1) | 115 bp | NA |
| AMA1 | PF3D7_1133400 | P0138_qPCR_F_AMA1 | P0139_qPCR_R_AMA1 | GGATTATGGGTCGATGGAAATTGTG | CATAATCTGTTAAATGTTGTTCATATTGTTTAGGTTGAT | (2) | 145 bp | NA |
| EBA175 | PF3D7_0731500 | P0142_qPCRF_EBA175 | P0143_qPCR_EBA175 | AATTTCTGTAAAATATTGTGACCATATG | GATACTGCACAACACAGATTTCTTG | (1,3) | 96 bp | After |
| EBA140 | PF3D7_1301600 | P0140_qPCRF_EBA140 | P0141_qPCRR_EBA140 | GCAAAATAAATGCAACAATGAATA | AACAAGGACCCGGTGAACTA | (1,3) | 80 bp | After |
| EBA181 | PF3D7_0102500 | P0146_qPCRF_EBA181 | P0147_qPCRR_EBA181 | GCGGGTAGTACAATATTAGATGATTC | TGTTGTGTGCTAAAATTATGTTCTTG | (3) | 107 bp | After |
| EBA165 | PF3D7_0424300 | P0154_qPCRF_EBA165 | P0155_qPCRR_EBA165 | ATTAAATCGTACATCACATACGCA | ACGCCCATCATGCACATT | (3) | 100 bp | After |
| RH1 | PF3D7_0402300 | P0124_qPCR2_F_RH1 | P0125_qPCR2_R_RH1 | GATAAAGAGCAAGAAAAACAACAAC | CATTACCTCTTCTTGATTTCTACCA | (1) | 105 bp | After |
| RH4 | PF3D7_0424200 | P0134_qPCR_F_RH4 | P0135_qPCR_R_RH4 | GAAATGACGCAATTCCCTCAAAAGA | GGTGTGTTTTATTTATATCATGTTGATTCTGTGA | (2) | 93 bp | Before |
| RH2a | PF3D7_1335400 | P0128_qPCR2_F_Rh2a | P0129_qPCR2_R_Rh2a | ATTAAACCTACAAAGCATGGTGATA | GATCTGTTCCTGATCTTTTAGTTGA | (1) | 124 bp | After |
| Rh2b | PF3D7_1335300 | P0132_qPCR2_Rh2b_F | P0133_qPCR2_R_Rh2b | TGACACTGATGAAAATGCTGA | TGTCCTTCTTTATTTCCCCC | (1) | 129 bp | After |
| RH3 | PF3D7_1252400 | P0152_qPCR_F_RH3 | P0153_qPCR_R_Rh3 | CACGAAAAATTCGAATAATGG | CCAATAGCAAATCCTGAAGC | (1) | 105 bp | After |
| RH5 | PF3D7_0424100 | P0118_qPCR_F_Rh5 | P0119_qPCR_R_RH5 | ACGAAGAATCAAGAAAATAATCTGACGTTACT | TGTTGAATGATCTTTAGCATTATTTGTTTTTATATTCTCTTT | (4) | 150 bp | NA |

**Reference**

1. Stubbs J, Simpson KM, Triglia T, Plouffe D, Tonkin CJ, Duraisingh MT, et al. Molecular Mechanism for Switching of P. falciparum Invasion Pathways into Human Erythrocytes. Science (1979) [Internet]. 2005 Aug 26;309(5739):1384–7. Available from: <https://www.sciencemag.org/lookup/doi/10.1126/science.1115257>

2. Nery S, Deans AM, Mosobo M, Marsh K, Rowe JA, Conway DJ. Expression of Plasmodium falciparum genes involved in erythrocyte invasion varies among isolates cultured directly from patients. Mol Biochem Parasitol [Internet]. 2006 Oct;149(2):208–15. Available from: <https://linkinghub.elsevier.com/retrieve/pii/S0166685106001721>

3. Blair PL. Transcripts of developmentally regulated Plasmodium falciparum genes quantified by real-time RT-PCR. Nucleic Acids Res [Internet]. 2002 May 15;30(10):2224–31. Available from: <https://academic.oup.com/nar/article-lookup/doi/10.1093/nar/30.10.2224>

4. Gomez‐Escobar N, Amambua‐Ngwa A, Walther M, Okebe J, Ebonyi A, Conway DJ. Erythrocyte Invasion and Merozoite Ligand Gene Expression in Severe and Mild Plasmodium falciparum Malaria. J Infect Dis [Internet]. 2010 Feb;201(3):444–52. Available from: <https://academic.oup.com/jid/article-lookup/doi/10.1086/649902>
