# Supplementary material for: Application of optical tweezer technology reveals that PfEBA and PfRH ligands, not PfMSP1, play a central role in Plasmodium falciparum merozoite-erythrocyte attachment
Source: PLoS Pathog. 2024 Sep 23;20(9):e1012041. doi: 10.1371/journal.ppat.1012041 (PMC11449297; doi:10.1371/journal.ppat.1012041)
Supplement: Attachment — Submitted filename: Review feedback response.pdf [file ppat.1012041.s025.pdf]

Part I - Summary
Please use this section to discuss strengths/weaknesses of study, novelty/significance, general execution and scholarship.

Reviewer #1: This paper reports on a technically highly challenging study investigating the molecular mechanisms governing red blood cell invasion by the major malaria causing parasite. It's innovative and provides important and interesting insights. Using a recently established optical tweezer assay in combination with inhibitors and genetically modified parasite lines the authors probe the entry of P. falciparum merozoite into red cells. The probe the attachment frequency and strengths of merozoites. Specifically, they provide evidence that not MSP1 but RH4 is important for strong attachment of the parasite to the red blood cell and that GYPA on the red cell surface also plays a key role.

Reviewer #2: Optical tweezers were used to manoeuvre human RBCs to pick up newly released P. falciparum merozoites and to bring the parasites to other RBCs. Merozoites adhere to RBCs prior to invading them and so by using the tweezers to 'pull apart' the RBC-merozoite-RBC sandwiches, the strength of merozoite binding could be inferred by measuring the stretching deformation of the RBCs which have well-established elastic properties. The biggest surprise of the work was that disruption of gene encoding the major merozoite surface protein MSP1, produced no reduction in binding strength suggesting MSP1 is not involved in RBC attachment. This runs counter to many years of work although recent genetic knockout experiments indicate MSP1 is probably more important for the egress of merozoites during schizont rupture. Further work with the knockouts of merozoite genes for EBA and RH proteins as well as treatments which block their corresponding RBC receptors indicates that EBA175 and RH4 might be the most important merozoite ligands for primary RBC attachment.

Reviewer #3: This manuscript by Kals and colleagues titled "Optical tweezers reveal that PfEBA and PfRH ligands, not PfMSP1, play a central role in Plasmodium falciparum merozoite-erythrocyte attachment" dissects in detail binding strength, attachment frequency and invasion capacity of Pf merozoites. The authors succeed in a clear dissection of the contribution of individual ligand-receptor pairs for RBC invasion. Whereas previously published studies had identified the role of individual ligand receptor pairs by analysing real time imaging of the invasion process, quantitation of this data was difficult. Still, the field had accepted that EBAs and RH parasite ligands were involved in strong interactions with the RBC receptors and facilitate the deformation of the RBC seen during real time invasion visualisation. Here the authors use optical tweezers to measure the binding strength of merozoites to their host cells both using wild type but also mutants and with that managed to determine and actually measure that EBA175-GPA and RH4-CR1 in particular are significantly contributing to the binding strength and with that invasion success of merozoites. Whereas the majority of the data presented in this manuscript is not surprising, the methodology used here gives confirmation and quantitation and validation to previously accepted truths.

Reviewer #4: Kals et al apply optical tweezers to examine the contribution of different malaria merozoite surface exposed antigens to binding of the erythrocyte. The data in this study provides highly relevant and useful information to the field on what proteins actually contribute to binding and what inhibitors may actually act against it. This

breaks the fields previous understanding which was largely based on indirect measures and speculation based on protein localisation. The technique, and therefore the study, has its limitations, but the authors provide a significant level of experimental detail to explore and mitigate these limitations as best as possible. The manuscript is mostly well written and the data completed to a high standard.

Part II – Major Issues: Key Experiments Required for Acceptance
Please use this section to detail the key new experiments or modifications of existing experiments that should be absolutely required to validate study conclusions.

Generally, there should be no more than 3 such required experiments or major modifications for a "Major Revision" recommendation. If more than 3 experiments are necessary to validate the study conclusions, then you are encouraged to recommend "Reject".

Reviewer #1: The study is well conducted and I don't have any experimental suggestions but the written part of the paper should be improved by condensing the text (suggestions below)

Reviewer #2: The work in the paper is very comprehensive and performed to a high standard and so no major additional work is required.

Reviewer #3: Overall, this manuscript is an important scientific contribution to the field of invasion biology of Plasmodium parasites with the authors making use of their unique ability to measure merozoite attachment strength using their optical tweezer setup.

Although no additional data has to be provided to support the major conclusions in this manuscript some of the authors interpretations especially around the function of MSP1 are unsubstantiated and unless the authors are willing to conduct further experiments should be removed.

1. Lines 249-250: Giemsa-stained smears or SybrGreen-labelled FACS based parasitemia counting of cDELPfMSP1 cannot determine whether there is an invasion defect with these parasites. Given the documented egress defect of this mutant cell line, the lack of ring stages post egress, cannot be contributed to an invasion defect but an accumulative growth defect.

The reviewer is absolutely right, the methods used at this specific point were to establish that the cDELPfMSP1 line had the expected difference in growth rate post rapamycin addition, not to establish whether it had an invasion defect specifically – the inclusion of the words “invasion rate” was an error. We have now adjusted the manuscript wording to make this more clear. It now reads:

Giemsa-stained smears post-rapamycin treatment showed a mean 2.3-fold reduction in parasitemia compared to DMSO-treated controls, **S5 Fig a and b**, in keeping with previously published data (45).

=> I was disappointed as the optical tweezer technology should allow the authors to manipulate individual cDELPfMSP1 merozoites from the stringy egress net and test

whether they are, despite their egress defect, still able to invade red blood cells, but this experiment has not been done here. This would allow the speculation of whether MSP1 plays a role in egress AND invasion, or ONLY in egress to be settled. 2. lines 588-589 in Discussion state that as ‘cDELPfMSP1did not show a significant decrease in attachment or detachment force this is providing evidence for a role of MSP1 in egress’. Although detachment strength in this study was correlated with invasion success, like RH5 and AMA1 come after the strong binding stages of invasion, the weak strength interaction of MSP1 might facilitate the subsequent engagement of strong interactors such as EBA175. Knowing that MSP1 function is involved in egress does not rule out in addition a function in invasion. As stated above, the authors have a unique tool to decipher egress from invasion phenotype of cDELPfMSP1parasites. Please use it.

We completely agree that it is formally possible that cDELPfMSP1 parasites could have both an egress phenotype and an invasion phenotype. However this manuscript is focussed on identifying the ligands that govern merozoite-RBC attachment strength, and we believe that the broader function of PfMSP1 in invasion is beyond its scope. Some of the co-authors on this manuscript are in fact using the conditional deletion line for additional detailed studies of the role (or not) of PfMSP1 in invasion more broadly and a manuscript describing the findings is currently being drafted (Lees *et al.,* manuscript in preparation). We have reworded the manuscript to clarify that our findings and interpretation relate specifically to the role of PfMSP1 in attachment strength, rather than making any statement about its role in invasion more broadly.

Regarding the statement that “weak strength interaction of MSP1 might facilitate the subsequent engagement of strong interactors such as EBA175”, if PfMSP1 interaction indeed facilitated other strong interactions in a significant manner, we would still expect to see a subsequent change in detachment force or attachment frequency when PfMSP1 is deleted, which we do not observe. It is formally true that we cannot rule out that there is some very weak interaction of PfMSP1 involved in attachment strength, the contribution of which would be below the sensitivity of the optical tweezer measurements. However we believe that the optical tweezer data does allow us to say PfMSP1 does not have a significant role in attachment, as has long been thought in the field. We have tried to make this distinction more clearly in the revised version.

Finally, the specific experiment proposed is intriguing, but it is one that we think would be technically very difficult. The throughput of being able to manipulate a single merozoite from the stringy egress net of the MSP1 null parasites is very low (typically just one or two merozoites per egress). The optical traps cannot be too close to the hemozoin crystals as they cause it (and any attached merozoites) to fly out of the field of view. Therefore, to manipulate merozoites, they have to be at the edges of the egress clump or one of the few that escape the clump. This would make manipulating enough merozoites to assess invasion extremely time-consuming. As stated above, the broader role of MSP1 in invasion is being explored in other work.

Reviewer #4: (No Response)

Part III – Minor Issues: Editorial and Data Presentation Modifications
Please use this section for editorial suggestions as well as relatively minor modifications of existing data that would enhance clarity.

Reviewer #1:
1 Title: Optical tweezers don't per se reveal anything, please rephrase. It's either their use or a particular type of use. Not sure you need to mention the technique in the title.

We have rephrased the title to make it clear that it is indeed the **use** of the optical tweezers that reveal the central findings, as suggested by the reviewer. As to whether to mention the technique at all in the title, we respectfully disagree with the reviewer's opinion here. We believe that it is the use of the optical tweezer technology that makes this study quite different to previous studies of invasion and think that inclusion of that factor in the title will make it easier for other researchers to identify this fact and find the publication on PubMed or other search engines.

21 please delete 'falciparum' as there are different species causing malaria or rephrase. It might be worth to mention that the different parasite species do have different invasion ligands.
The manuscript text has been amended as suggested.

29 not sure what the authors mean with 'phenotypes', maybe processes?
Manuscript wording changes as suggested, both here and in other places where attachment phenotype was used.

29-37 the abstract could in my view be written less defensively
The abstract has been rewritten to emphasise the central findings more emphatically as suggested.

47 'whilst using' – maybe better: by combining it with… or by probing a set of…
The manuscript text has been amended as suggested.

57 'merozoite egress from the liver' and '<60 sec'– merozoites are shed within merosomes and rupture in the lung, I don't know of any study that quantified how quickly this happens, a minute would be a good estimate but no need to make a statement here that might be wrong.
The manuscript text has been amended as suggested.

73 citation 11 might be replaced with the more recent work showing that the parasites go in with the wider end. At least this should be added (PMID: 34819379).
Reference added to the manuscript as suggested.

97 I suggest to rephrase this sentence. 'very low' is not quantitative and some of the assays are rather high throughput in comparison to other assays. Maybe better to state that video microscopy is passive observation. This then allows a more elegant bridging

to the optical tweezer assays. The following paragraph would benefit from not focusing solely on red bloo cell invasion but on the use of laser tweezers in general and then focus on the specific use case.
The manuscript text has been rephrased as suggested.

99 Again, it's not about quantitative, it's about being able to manipulate
The manuscript text has been rephrased as suggested.

140 s15 video, should that not be video1?
The numbering of the videos changed so that they are ordered with the supplementary figures in the order that they appear in the text.

157, 563 use 's' instead of 'sec'
The manuscript was updated to consistently use s as suggested.

165 please always only use two digits, e.g. 24 instead of 24.2
The number of significant figures for all numbers has been changed to 2 or an appropriate number for the error in the measurement.

230-355 to enhance readability, the text could be compressed by not repeating details from the introduction and move technical issues to the materials and methods. Also for following pages of the results, it reads in parts more like a PhD thesis than like a paper.
We have attempted to reword and move technical issues as suggested.

In my view figure 4 and most of the associated text could go to supplement as it adds little as minor changes in mRNA might not at all be reflected on the protein level. It distracts from a beautiful biophysical study.
We thought that qRT-PCR data was important – it is widely reported in the literature that transcription of PfEBA and PfRH ligands is variable, and we thought it critical to establish whether compensatory expression changes could be obscuring some phenotypes. Our data, comparing multiple lines, suggests this is not the case and emphasises that some genes are more variable in expression pattern than others.

However, we take the reviewer's point, that the emphasis of the paper is on biophysics. We have, therefore, condensed the text discussing the qRT-PCR results, moved the original Figure 4 to the supplement and instead added a single sub-panel summarising the expression data to the new Fig 3.

539 effectiency ?
The manuscript has been corrected.

541 However,… I am lost in this sentence, please rephrase
This section of the manuscript has been rephrased.

Is there anything known about attachment strength and frequency from other invasive parasite stage such as ookinetes or sporozoites or from T. gondii? If yes, please put your study into perspective. For those parasites optical tweezers have also been used.
To put the mean detachment force of the wild-type (3D7 - 35.9 ± 3.1 pN vs NF54 24.2 ± 1.5 pN) measured here into context, a typical single-molecule interaction ranges from ~1.7 pN for actin-myosin to ~160 pN for biotin-streptavidin (Bongrand, 1999). Interestingly, the forces of merozoites attaching to erythrocytes measured here are about 10 times lower than the forces measured of RBCs binding to iRBCs in rosettes (44nN) (Nash et al., 1992). These details were added to the manuscript to add more context.

Optical traps have also been used to look at *Plasmodium berghei* sporozoites, but these measurements are not directly comparable to ours as binding was not between two cells, sporozoites adhesion to glass slides was quantified (force greater than 24.6 ± 4.3 pN) (Hegge et al. 2012) and the force a sporozoite could pull glass beads (forces in range 70 to 190 pN) (Moreau et al. 2021). In addition, optical tweezers have been used to assess the force with which *Toxoplasma gondii* could move a microsphere attached to its surface (Stadler et al 2017). We agree, however, that these are all useful comparators for the reader, even if the details of the assays are different, and we have updated the Discussion to include them as suggested.

A number of references are cited twice and should be merged
The references have been checked, and any duplicates have been removed.

Reviewer #2:
1. It appears that the citations in the text to the figures do not always match up indicating the text and figures maybe from different drafts of the paper. These were FigsS3, S5 and S6, Figs 2 and 5. The paper needs to be very carefully checked before resubmission.
We have gone through the manuscript and double-checked all of the references to figures to try and ensure that they are now correct.

2. I am surprised heparin treatment was not assayed using the optical tweezers as merozoites still weakly attach to RBCs and herparin thought to interfere with MSP1 binding.
Heparin has a strongly inhibitory effect on invasion, but the question of how specific this inhibition is for PfMSP1 is not completely clear. The strongest evidence for heparin specifically interfering with PfMSP1 binding, as far as we are aware, is based on protein extracts from *P. falciparum* schizonts being passed over heparin-coated beads and showing $PfMSP1_{42}$ and $PfMSP1_{33}$ binding via western blot (Michelle J. Boyle *et al.*, 2010). However, only three other proteins other than PfMSP1 were assayed for binding, so it is possible that heparin could also affect other molecular interactions. We did, however, collect some preliminary data with heparin; it affected the attachment frequency but had no significant effect on detachment force (only 4 measurements). Heparin was also one of the original conditions tested by Crick et al in the first optical

tweezer methods paper, and they reported reduced detachment force, but this was generated from only 2 measurements and used a higher heparin concentration and a different wild-type line, ITO4.

Given the very large number of conditions tested and the amount of time each one takes (optical tweezers are not high throughput, and repeats take multiple weeks), we simply did not have the resources to test everything. As we had access to a much more specific test for PfMSP1 function, a conditional knockout line, we chose to focus our time on this line.

Preliminary heparin data

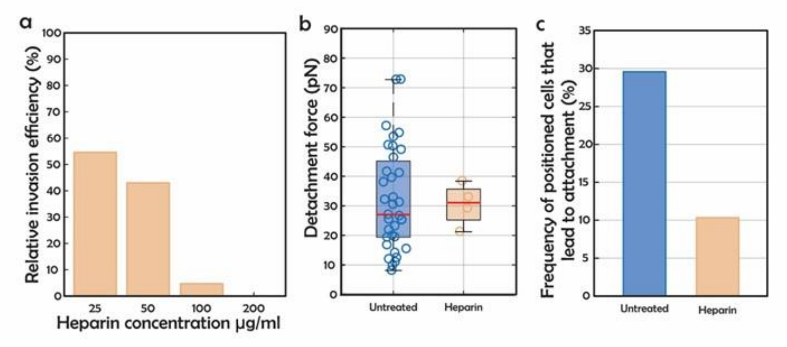

Data collected by Crick et al.

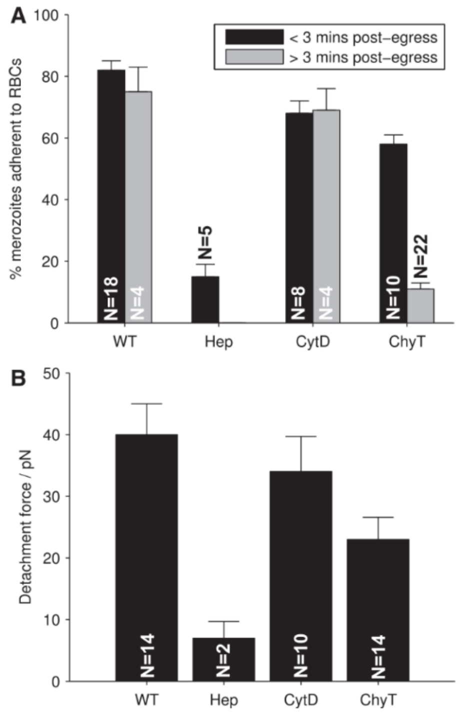

3. Why were EBA175 knockout parasites not tested? EBA175 is known to bind GYPA and this interaction was blocked with anti-GYPA IgG but what if EBA175 binds to other receptors or the antibody does not completely prevent EBA175 from accessing GYPA?

Prior to the reviewed manuscript submission, we had issues making the KOPfEBA175 line in the NF54 background. During the process of review we have now successfully made this line, and tested it as suggested. As noted in the manuscript, deletion of PfEBA175 alone had no significant effect on detachment force, suggesting that the anti-GYPA antibody is having a broader effect than only targeting the PfEBA175/GYPA interaction. We also tested a second KOPfEBA175 line, made in the Dd2 background which is thought to rely much more heavily on GYPA-EBA175 interactions for invasion. Deletion of PfEBA175 in this background actually increased attachment strength, which is perhaps explained by the fact that this deletion has been shown to significantly increase expression of PfRH4. There is therefore a consistency between the fact that deleting PfRH4 in NF54 decreases attachment strength, while deleting PfEBA175 in Dd2, which is known to increase PfRH4 levels, increases attachment strength, and suggests that PfRH4 may play a particularly important role in attachment. We do not believe that it is the sole contributor however, given the impact on neuraminidase. Finally, we combined the Dd2KOPfEB175 line with anti-GYPA antibodies, and found that even in the absence of PfEBA175, anti-GYPA could still decrease attachment strength.
This additional data has now all been added to the manuscript, and we believe adds further depth to our finding – we are grateful to the reviewer for suggesting this avenue.

4. The difference between the effects of R1 peptide which blocks AMA1 and the AMA1 knockout merozoites is noted. R1 increased the strength of merozoite binding and AMA1 knock out did not have an effect. Are the authors aware of https://doi.org/10.1007/s00018-023-04712-z where AMA1 was suggested to have a second receptor apart from RON2? Could the second interaction have kept merozoites engaged with the RBCs increasing the detachment force?
We were not aware of this paper, and we thank the reviewer for pointing it out. We have now included reference to this paper in the Discussion and agree it could provide one possible explanation for the higher attachment force seen when the R1 peptide was used.

5. Line 138. Check 'egressed so viable merozoite'
The manuscript has been corrected.

6. Line 156-157. Multiple names for seconds being used ie, sec and s.
Manuscript updated to consistently use s.

7. Line 165. I think I know what "attachment frequency per egress" means but please clearly define.
The manuscript was updated to define the attachment frequency per egress in the previous paragraph as suggested.

8. Line 175. "p=0.0000" Really?
Updated to p= ≤0.0000, which is the result of the ttest.

9. Fig S1B. Y-axis should be "Detachment Force"
Figure updated.

10. Line 219. “cells that lead to attachment.” Attachment to what?
The wording of the legends in the manuscript has been updated throughout. “The attachment frequency is calculated throughout as the mean of the frequency measured per egress.”

11. Line 317. Should be “anti-EBA140”?
Manuscript updated.

12. Fig 2 legend. Text for “d and e” is missing.
Manuscript updated.

13. Line 378 “0.952 when including them.” Should be excluding?
Manuscript text corrected.

14. Line 511. Fig 4 should Fig 5?
Manuscript updated.

15. Line 572. Could RBC age contribute to the variability observed?
Yes, variation in RBC age within a sample could result in a change in receptors exposed on the erythrocyte surface, which could in turn affect the measured detachment forces. We have added this possibility to the manuscript as suggested.

16. Line 598. “The binding of several of the PfEBA175, PfEBA140…..” Do you mean ligands or knockout parasites?
Manuscript text corrected.

17. Line 678 NaOH
Manuscript text corrected.

18. Line 1097. Fig S3. End of legend discusses NF54 detachment data that is not in the figure.
A sub-panel was added with this data to correct this oversight.

19. Line 1113. WR99210
Manuscript text corrected.

20. Line 1145. “..a little clumpy..” Use more accurate description that matches Table.
The wording of the legend was changed to make it more consistent with the table.

Reviewer #3:
In Lines 190-192 Fig S3 the authors point out that merozoite polarity does not significantly affect detachment score. Do the authors find this not surprising? Especially given that the strongest detachment score is measured with apically secreted ligands such as EBA175? Could the authors elaborate a bit more on this?
We have rephrased the section of the manuscript to elaborate and clarify our interpretation. We were unable to visualise the side of detachment based on a

fluorescence marker, so instead, we looked at whether detachment was more likely to occur from the first or second erythrocyte the merozoite attached to. Our logic here was that if reorientation preferentially brings the apical (invasion) end in to contact with the first erythrocyte contacted, then detachment from the first erythrocyte would likely be from the apical end. There was indeed a slight preference for detachment to occur from the second erythrocyte (67.7%), but there was no significant difference in detachment forces from the first/second erythrocyte detachments. This is not entirely conclusive but does indicate that it is likely not a strong preference as to which side of the merozoite detachment is occurring (ie detachment does not always happen from the second, presumably non-apical, end of the merozoite). In addition, when we plot detachment force against time post-egress, we do not see a correlation. The gradient of ligands is thought to change after egress as proteins secreted from apical organelles diffuse around the merozoite; if the gradient was having an impact on detachment force, then we would expect to see a correlation between detachment force and time post-egress, which we did not observe. We, therefore, have no strong evidence that polarity affects detachment force, but our measures are indirect, not direct, so we have modified the strength of our interpretation accordingly.

This is perhaps not what might be expected based on the pictures in the literature of highly polarised merozoites. However, whilst ligands such as members of the PfEBA/PfRH family are secreted at the apical end of the merozoite, they diffuse rapidly around the merozoite surface, which likely means strong attachment can occur at any point of the merozoite. We have looked into the literature regarding the role of the gradient of ligands in the invasion. The evidence for its role in invasion seems to be primarily based on modelling (Dasgupta et al. 2014), and this model does not encapsulate more recent observations based on video microscope data (such as that the broader end of the merozoite is the side reorientation occurs to, that the myosin motor is needed for deformation and that membrane wrapping occurs and then relaxes before the merozoite then pushes into the RBC). We think more experimental evidence is needed to understand the actual density of receptors over the merozoite surface post-egress and the number of receptors that engage in erythrocyte binding to properly understand how the gradient of ligands impacts attachment. This is however a fascinating area for further work, and we have tried to represent the reviewer’s questions in our rewording.

2. It would be helpful if the authors had quantitated their gene excision/invasion efficiencies (lines 247-248). Given antibodies have been used by the authors to assess whether excision has occurred (IFAs), the excision rate of the DiCre-mediated experiment could have easily been determined.

We have now quantified the excision efficiency of the rapamycin-treated samples used on the IFA data, which showed a 94.32% excision rate. We have added this to the manuscript.

Although the overall data in this manuscript is important, the manuscript in its current form is full of errors/inaccuracies and needs a significant and careful re-assessment by the authors.

We have been though the manuscript carefully and have tried to correct any errors/ inaccuracies.

• At least in the pdf provided to the reviewers the readability and clarity of figures needs improvement. Firstly, the median is often not visible, the significance comparisons are hard to make out, the y-axes have no clear lines to determine where different % start and it would be helpful here to include not only lines at intervals of 10, but also at half of that.

The median line has been made bolder so that it is easier to see. The significance comparisons have been made slightly bigger to make them clearer. The background of the graphs has been made slightly darker and the interval lines made wider to make it easier to determine the different intervals. However, sublines were not added as we felt that they made the small graphs too busy and hard to interpret. If the reviewer feels they are still necessary after seeing the updated version, then they can definitely be added)

• In other Figures such as Fig 3 it would be helpful to have the main data figure central and move sub-panels a) and b) to the supplementary as these are not data and only distract. The summary figure 6 however is very helpful and informative for the reader.

The region of the gene replaced by the selectable marker is important to the understanding of the qPCR data so we felt it was important to keep sub-panel a) as part of the main figure but we have removed sub-panel b).

By lines/figures:

Lines 172-174: PEMR should read as dividing the ring parasitemia by the parasitemia the day before and not the other way around.

The manuscript has been corrected.

FigS4 WR99210, not WT9921

The manuscript has been corrected.

Lines 1144-1146- S5Fig legend: grammar/spelling

The manuscript has been corrected.

Lines 1149 S5Fig which statistical testing was used here? “a test”

The manuscript has been updated, and the mistake corrected so it now reads ttest was used.

Lines 254 – 256: referring to cDELPfMSP1attachment strength measuring but does not refer to a figure where these data should be verifiable.

The manuscript has been updated.

Lines 271 -272: “efficient gene excision with no band, indicating the presence of unedited parasites in the rapamycin-treated samples”? Obviously, this is not what is meant here, rather the opposite.

The manuscript wording has been changed to make the meaning clearer.

Lines 272-273: There is no panels f or g in S5 Figure. And where do the 1.43% invasion efficiency data come from? In S5 Fig that invasion efficiency is listed as 0% - 4.3%.
Manuscript updated to S5c. The 1.43% is the mean of the three repeats of invasion efficiency measured.

Fig S6 c) were the authors not surprised that with 40uM of R1 peptide the parasites still achieved ~20% of their normal invasion rate? Are these 20% real, living parasites?
Parasitemia was assessed using flow cytometry to count the percentage of erythrocytes that were positive for DNA (detected with Sybr green dye), indicating the presence of a parasite. It is not possible to distinguish with flow cytometry between rings and erythrocytes with merozoites stuck on the outside of the erythrocyte as they were unable to complete invasion. We had the same question when doing the experiments and so imaged the sample at the same time as the flow analysis on an EVOS microscope. In both the untreated control and the sample treated with 40uM R1 peptide, we saw both rings (star-shaped DNA signals) in erythrocytes as well as merozoites that appeared to be stuck on the outside (control example 1 and R1 peptide example 3). This indicates that some, but not all, of the 20% parasitemia signal present with the 40uM R1 peptide is rings; we did not, however, maintain the culture to see if the parasites could develop further. These measurements are in keeping with previously reported invasion rates; 21um R1 peptide gave an invasion rate of around ~10% of the control in 3D7 (Lamarque et al. 2011). We have added this explanation to the text.

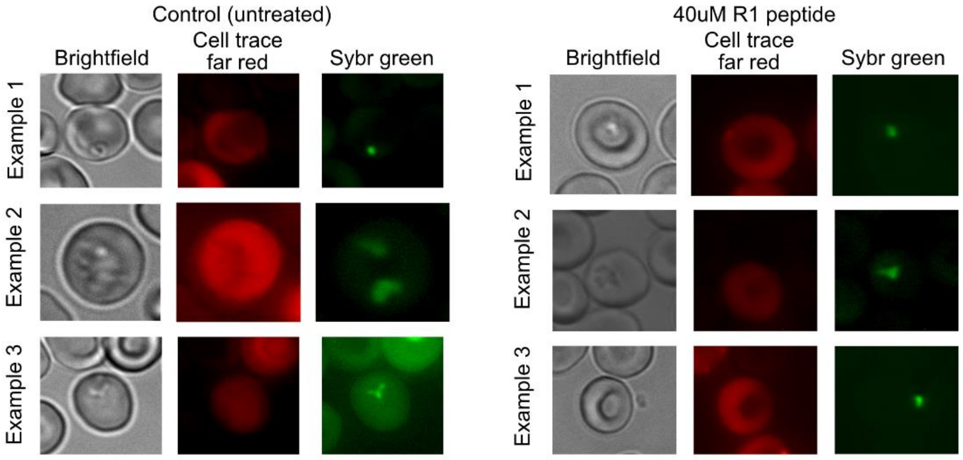

Line 296: “Neuraminidase treatment reduced invasion by nearly half..” where is this data displayed?
The manuscript has been updated as suggested.

Line 298: Fig 2d, not 2e
The manuscript has been updated as suggested.

Line 309: “anti-GYPA invasion was 55.5%...” in which figure is that data shown?
The manuscript has been updated as suggested.

• Line 310 Fig2d
The manuscript has been updated as suggested.

• Line 312 Fig 2d
The manuscript has been updated as suggested.

• Line 321 Fig 2d. As no Fig 2e or f exists
The manuscript has been updated as suggested.

Line 333 insert Figure number you show this anti-Basigin data
The manuscript has been updated as suggested.

Line 337 Fig 2d
The manuscript has been updated as suggested.

Line 347 Fig 2d
The manuscript has been updated as suggested.

Line 354 S5 Fig e does not exist
The manuscript has been updated as suggested.

Line 374 is not clear- please spell out "there was a weak correlation…" (between which two variables?).
The manuscript was updated to specify what the correlation was between, and the colours were changed in the figure to make it clearer.

S7 Fig b) is unclear. According to the diagram I would expect C,D only to amplify with wt locus primers; whereas A and B should not. Panel b only confuses. Instead list one primer pair number in the graphic in a) and then go to PCR data images.
Panel b had been removed, and the mistake in the numbers in panel a was corrected.

Fig 4: has no panel f although that is listed in legend
The manuscript legend has been updated to remove f.

S10 Fig (line 1205) b-c should read a-b as no panel c exist
Manuscript text updated.

Line 511: Fig5, not Fig4
Manuscript text updated.

Fig 6 correct spelling mistakes
The spelling of merozoites was corrected.

***Reviewer #4**: -In general, the manuscript is well written, but there are quite a lot of small errors, some of which I put further down. The manuscript would benefit from a good round of editing.*

-An EBA175 knock-out parasite is briefly mentioned several times in the manuscript, but does not actually seem to be used for the experiments. Given the interpretation

around the importance of EBA175 for binding through GLYA experiments, I find this surprising and it would obviously improve the manuscript if this data was available and included. This would greatly strengthen the support for EBA175 being important for binding. However, it is not included and so I find this distracting and I am not sure why the data is there. If the data was done then it would be best to include it. If it is to be used in another paper then it would be best to state this and remove any reference to the EBA175 knock-out where it is not needed. At this stage, as written, it does not seem to be needed.

See response to Reviewer 2

-The measurement of Frequency of Attachment is a useful measure as merozoites can sometimes attach to multiple cells or multiple times to a cell before invading. In this study, these measures were done on wildtype parasites. Could the authors speculate on whether surface exposed merozoite antigens could have a role in this multiple attachment potential and whether this could be explored using knock-out lines? Although most knock-outs used in this study show no change in erythrocyte binding, I wonder whether the ability to reattach might also be important in a flow environment.

This is indeed a fascinating question. We did not set out to try and measure attachment forces for the same merozoite many times, but we did try to reattach a merozoite after detachment if it was well positioned to do so. Analysing that data for the wild-type conditions revealed no change in detachment force even after the same merozoite had been used in many attachments. This suggests that either ligands can bind reversibly and be reused multiple times, or that the merozoite has a large pool of ligands that can attach. However, there were not enough examples of multiple measurements of detachment force for the same merozoite in our data to allow comparisons between knockout lines.

-Why would GAP45 impact on initial attachment? Typically, crude measures of disrupting the invasion motor by CytoD lead to disruption of entry. The paper cited did not report any change in merozoite binding to erythrocytes (whether they looked at this is not mentioned), only in deformation. Biologically, I don't really see why the glideosome would be linked to attachment frequency. Can the authors discuss this observation and possible mechanism some more.

GAP45 null parasites had a lower attachment frequency; lower attachment frequency could be caused by a lower initial attachment rate or a higher detachment rate; we cannot distinguish the two. Our data does not provide any further explanation for the mechanism, but we could speculate. One explanation could be that in the absence of a functional glideosome, merozoites are more likely to detach because without deforming the erythrocyte membrane they cannot proceed to the later steps of invasion (and initial attachment is the same). We have added this hypothesis to the manuscript.

-For the CR1 data, it might be worth exploring the limitations of other inhibitors (soluble CR1, antibodies) given the need for neuraminidase treatment to see a growth defect.

This would provide a rationale for why these additional steps may not have been taken in this study to explore Rh4 and CR1.

We explored an alternative anti-CR1 antibody, but we were only able to find one commercially available polyclonal antibody that did not contain sodium azide, a polyclonal antibody ABIN3215212 from the company Antibodies Direct. We ran one repeat to test the invasion rate in both untreated and neuraminidase-treated erythrocytes and one optical tweezers assay. No significant effect was seen in invasion rates, detachment force or attachment frequency, as shown below. Given no effect was seen, we again could not validate that the antibody was working. We have not explored the use of soluble CR1 as there was simply no time to do so, given the low throughput nature of the tweezer assay and the large number of lines and conditions being used. The purpose of this manuscript was to carry out a broad survey of multiple invasion steps/proteins – having identified the PfEBA/PfRH family in general and PfRH4 in particular as central to attachment, we plan to follow this up in more detail in future work.

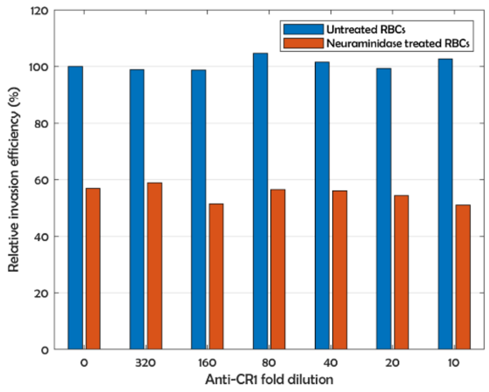

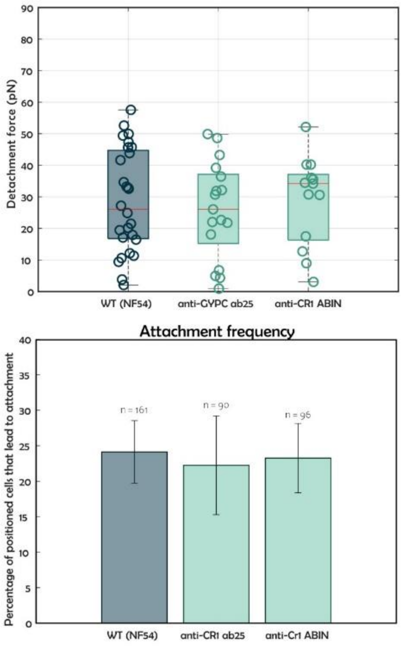

As I understand the data, the neuraminidase treatments lead to a 15-fold loss in attachment frequency, but only a 2 fold loss in invasion. This does not seem possible. Can the authors speculate why? Could this indicate a difficulty in using the frequency measure or how that is assessed?

We were also surprised by this, and think there are several possible explanations. The attachment frequency measures the likelihood of attachment of a merozoite to two erythrocytes when it is positioned between them. If the ability to attach to one erythrocyte had halved, then the ability to attach to two erythrocytes would be even lower. In addition, within the population of erythrocytes in a sample, there could be variation in receptor density on the surface. Therefore, some erythrocytes may not be able to attach at all, which, when we are testing attachment to two erythrocytes, could also amplify the effect. It is also possible that when we measure attachment, we do not see very weak attachments, as the attachment must be strong enough to be visually detectable by video microscopy, whereas some of these weaker attachments

could be strong enough for invasion to occur in prolonged *in vitro* culture conditions. We have added these potential explanations to the manuscript.

-The current dataset suggests that EBA15 and Rh4 are linked to attachment, but not the other EBAs and Rhs. Yet these proteins are considered to have redundancy in function. Do the authors consider that redundancy does not include the function in attachment? Or is this an indication of the limits of the knock-outs where it is known that some lines naturally do not rely on certain EBAs and Rhs and so knock-outs tend to have minimal impact. I think it would be worth clarifying this beyond an interest in double knock-outs etc.

Based on our data, we cannot determine if some ligands form weaker interactions than, for example, PfRH4 (which could be dominant if expressed) or if some ligands are not preferentially utilised in the NF54 strain tested, so they have a minimal role in attachment in this specific genetic background. A combination of investigation of knock-outs made in other wild-type backgrounds and double-knock-out lines would help address this further in the future. We have updated the relevant section of the discussion to clarify this.

-Figure 2A: spelling of merozoite.

Figure updated.

-Any reason why both 6 and 8 cycles were used for PEMR in the study?

We wanted to ensure that the samples were split into blood from 3 different donors over the course of the experiment to introduce biological replicates. Based on when blood was available from the blood bank, this took 8 cycles for one experiment (there were severe shortages at the time the experiment was run, so we were limited in when we received blood samples) but only 6 cycles for the other experiment.

-Figure 6: spelling of Merozoite (x2)

The manuscript has been corrected.

-Units: By convention, there should be a gap between the number and the SI unit. Throughout the text some measures have a space and some do not.

The units in the manuscript have been updated to include spaces consistently.

-Below I provide some passages where I suggest the wording could be improved or words removed/added (this is not exhaustive so some more review would be suggested).

Line 138: used to manipulate a newly egressed so viable merozoite and position

Manuscript wording updated.

Line 187: Line starting ‘Merozoites are polar cells..’ and the subsequent paragraph. I found the concepts explored here difficult to follow as written. If is clearer in Fig S3. I suggest making this passage clearer.

The text of the manuscript has been rephrased to hopefully make it clearer.

Line 296: There ‘was’ also significantly reduced attachment.

Manuscript text corrected.

Line 297: for neuraminidase-treated erythrocytes (1.4 ± 0.5%), 'which was' significantly different to NF54 (rank-sum p=0.0001, Fig 2e);
Manuscript text corrected.

Line 319: of levels observed 'in' the absence of the antibody.
Manuscript text corrected.

Line 424: For all genes, this insertion was made within the predicted erythrocyte binding domain for all genes other than PfRH4 (repeated text).
Manuscript text updated to removed repeat.

Line 429: receptors were known. (where?).
Manuscript updated.

Line 539: effectiency
Manuscript updated.

Line 543: as if invasion is blocked after attachment has occurred, then it is expected that the invasion rate would be reduced.
This section of the manuscript has been rephrased.

Line 544: completion. (completeness??)
The text in this section of the manuscript has been updated.

Line 607: face of increase shear. (increasing??)
Manuscript updated.

Line 647: role of PfMSP1 not in. (is not??)
Manuscript updated.

Line 654: the NF54 background, we used, the interactions of PfEBA175 and PfRH4 654 appear. (too many commas?)
Manuscript updated.

Line 720: erythrocytes the coverslip. (to the??)
Manuscript wording corrected.

Line 732: the erythrocyte length (Lo) of each erythrocyte. (probably don't need the first erythrocyte). It is also worth looking at the wording at this section. The sentence just mentioned implies that measures were made for each erythrocyte. The final sentence is not clear what it means: If two erythrocytes were stretched, then only the detachment force was recorded.
Manuscript wording changed to improve clarity.

Line 735: $F = k\Delta L$,. (comma?)
Manuscript corrected.

A couple of times: FSCA vs FSC-A. A couple of your flow cytometry methods are quite similar. Could be worth considering having a single separate method?
Whilst a couple of the methods are similar the steps for the three assays that used the flow cytometry were different depending on the application and we felt to ensure clarity for anyone trying to replicate the method it was important to be clear for each application. We have however removed duplicated steps of the initial gating.

Line 827: after the invasion widow. (window?)
Manuscript wording changed.

Line 852: The selectable was. (wording needs changing???)
Manuscript wording changed.

Line 880: while the R1 peptide (VFAEFLPLFSKFGSRMHILK) (GenScript at 98% purity). (wording needs changing???)
Manuscript wording changed.

There is a repeat in text between acknowledgements and financial disclosure. May not be necessary?
As they Acknowledgement and financial declaration do not appear next to each other in the final manuscript we included these details in both but can remove the duplicate information if preferred.

Line 1070: detachment force of merozoite-erythrocyte detachment force. (repeated text could be reworded?)
Manuscript text corrected.

Line 1088: are summaries. (summarised??)
Manuscript text corrected.

S3Fig 1: (c-d) Show……. Then (d) shows. (This happens a few times. Capitals or not for all?
The manuscript has been corrected so that all first letters are capitalised, and all the legends have been checked for the same mistake.

Line 1145: The table table. (repeated?)
Manuscript text corrected.

Line 1150: (c) cΔPfAMA1. (d) cΔPfGAP45. (These descriptions are a bit brief. Worth expanding?).
Manuscript description expanded.

-Any reason why the downstream (truncated) region of PfRH4 was not amplified when checking knock-out?
Several qPCR primer pairs were tested for each gene, using examples in the literature wherever possible. Two previously published sets of primers were tested for PfRH4, one downstream and one upstream of the PfRH4 disruption region. The downstream version of PfRH4 primers was less sensitive when optimising on genomic DNA with low Cq values at low concentrations than other primers being tested. The upstream

version was better when tested on a concentration gradient, so we selected that primer pair.

PLOS authors have the option to publish the peer review history of their article (what does this mean?). If published, this will include your full peer review and any attached files.

If you choose “no”, your identity will remain anonymous but your review may still be made public.

Do you want your identity to be public for this peer review? For information about this choice, including consent withdrawal, please see our Privacy Policy.
Reviewer #1: No
Reviewer #2: No
Reviewer #3: No
Reviewer #4: No
