# Supplementary material for: Application of optical tweezer technology reveals that PfEBA and PfRH ligands, not PfMSP1, play a central role in Plasmodium falciparum merozoite-erythrocyte attachment
Source: PLoS Pathog. 2024 Sep 23;20(9):e1012041. doi: 10.1371/journal.ppat.1012041 (PMC11449297; doi:10.1371/journal.ppat.1012041)
Supplement: Attachment — Submitted filename: Reviewer feedback.pdf [file ppat.1012041.s026.pdf]

Reviewer's Responses to Questions

Part I - Summary
Please use this section to discuss strengths/weaknesses of study, novelty/significance, general execution and scholarship.

Reviewer #1: As stated in my initial review, this is an interesting study that needed essentially a lot of textual improvement, which the authors now provided.

Reviewer #2: (No Response)

Reviewer #3: (No Response)

Reviewer #4: No further comments.

Part II – Major Issues: Key Experiments Required for Acceptance
Please use this section to detail the key new experiments or modifications of existing experiments that should be absolutely required to validate study conclusions.

Generally, there should be no more than 3 such required experiments or major modifications for a "Major Revision" recommendation. If more than 3 experiments are necessary to validate the study conclusions, then you are encouraged to recommend "Reject".

Reviewer #1: none

Reviewer #2: (No Response)

Reviewer #3: (No Response)

Reviewer #4: No further comments.

Part III – Minor Issues: Editorial and Data Presentation Modifications
Please use this section for editorial suggestions as well as relatively minor modifications of existing data that would enhance clarity.

Reviewer #1: there are some small typos that can be easily corrected in proof stage

The manuscript has been updated to try and correct any typos found.

Reviewer #2: The authors have satisfactorily address most issues of concern except for Fig S13c. Fig S13c is mentioned 2x on page 18. There is no S13c so change to a or b.

The incorrect figure number indicated has been corrected.

Reviewer #3: (No Response)

Reviewer #4: No further comments.
